# Severe acute malnutrition and infection

**DOI:** 10.1179/2046904714Z.000000000218

**Published:** 2014

**Authors:** Kelsey D J Jones, James A Berkley

**Affiliations:** 1 KEMRI—Wellcome Trust Research Programme, Kilifi, Kenya; 2 Department of Paediatrics and Wellcome Centre for Clinical Tropical Medicine, Imperial College, London, UK; 3 Centre for Tropical Medicine and Global Health, Nuffield Department of Clinical Medicine, University of Oxford, UK

**Keywords:** Malnutrition, Children, Infection, Antibiotics, Pneumonia, Sepsis, Diarrhoea, HIV, Tuberculosis, Measles, Urinary tract infection, Malaria

## Abstract

Severe acute malnutrition (SAM) is associated with increased severity of common infectious diseases, and death amongst children with SAM is almost always as a result of infection. The diagnosis and management of infection are often different in malnourished versus well-nourished children. The objectives of this brief are to outline the evidence underpinning important practical questions relating to the management of infectious diseases in children with SAM and to highlight research gaps. Overall, the evidence base for many aspects covered in this brief is very poor. The brief addresses antimicrobials; antipyretics; tuberculosis; HIV; malaria; pneumonia; diarrhoea; sepsis; measles; urinary tract infection; nosocomial Infections; soil transmitted helminths; skin infections and pharmacology in the context of SAM. The brief is structured into sets of clinical questions, which we hope will maximise the relevance to contemporary practice.

## INTRODUCTION

Children with SAM are classified as ‘complicated’ if they have clinical features of infection or metabolic disturbance, severe oedema or poor appetite. Children with ‘uncomplicated’ SAM are clinically well, alert and have retained their appetite. They should usually be managed as outpatients where suitable services with access to ready-to-use therapeutic food (RUTF) exist, whilst children with complicated SAM should be managed as inpatients.^2^ Thus, much of the information in this brief on infections relates to inpatient care, since children with features of infection usually require admission. However, an understanding of how children with infection present and initial management strategies are crucial for health workers dealing with new presentations of SAM, and for those dealing with complications and reassessing children for poor response to treatment during rehabilitation.

The term ‘WHO guidance’ throughout refers to practices outlined in the 2013 WHO Guideline: updates on the management of severe acute malnutrition in infants and children (http://apps.who.int/iris/bitstream/10665/95584/1/9789241506328_eng.pdf) and the proposed 2013 revisions to the WHO manual on management of severe acute malnutrition. For general paediatric care, we refer to guidance outlined in the 2005 WHO Pocketbook f Hospital Care for Children. The Pocketbook has since been revised (http://www.who.int/maternal_child_adolescent/documents/child_hospital_care/en/index.html) and, where possible, this document takes into consideration the proposed revisions. We have tried to highlight where we are providing ‘opinion’ rather than ‘evidence’.

## ANTIMICROBIALS

### Do children with SAM but no signs of infection need antibiotics?

Severely acutely malnourished children have traditionally been treated with broad-spectrum antibiotics at presentation even in the absence of overt infection.^3^ The rationale for this is that (i) malnourished children frequently have bacterial infections (including bacteraemia); (ii) diagnosing infection in malnourished children is difficult because clinical manifestations of infection (e.g. fever) may not be apparent; and (iii) malnourished children have bacterial overgrowth in their small bowel. However, while this approach has a sound and rational basis, there has been very little evidence of its effectiveness until recently. In 2013, a study from Malawi powerfully demonstrated the importance of antibiotic provision to children with SAM without clinical features of infection: 2,767 children with SAM eligible for outpatient care and aged 6–59 months were randomised to 7 days treatment with oral amoxicillin (80–90 mg/kg/day), cefdinir (an oral third-generation cephalosporin at 14mg/kg/day), or placebo. The 12-week mortality rates were 4.8% (amoxicillin), 4.1% (cefdinir), and 7.4% (placebo), giving relative risks for mortality for placebo compared to amoxicillin of 1.55 (95% CI 1.07-2.24), and for placebo compared to cefdinir of 1.80 (95% CI 1.22–2.64). Differences in mortality and recovery between the amoxicillin and cefdinir arms were not statistically significant.^4^

This substantial mortality reduction in children without overt infection and eligible for outpatient treatment in a rural area suggests that the provision of antibiotics should remain routine (and critically important) in all settings where SAM is managed outside of hospitals, regardless of the presence of features of infection. In this population the rates of HIV and kwashiorkor were high, but we consider that the burden is on demonstrating with trial evidence the safety of deviating from this management strategy (which is currently recommended by WHO) in other populations and settings, rather than considering that implementation can be postponed.

### Which antibiotic is most appropriate for outpatient care?

Ideally, choice of antibiotics should be guided by knowledge of which organisms need to be treated and their likely resistance profile, informed by microbiologic assessment for reference in cases of treatment failure and to provide local population resistance data. However, in areas where malnutrition is common, microbiologic services tend to be weak and treatment choice is influenced by cost, availability and ease of administration, as much as by effectiveness. In practical terms, while the Malawian study described above suggested possible advantages of cefdinir over amoxicillin, in the absence of further more definitive evidence of increased efficacy compared to readily available antibiotics, oral third-generation cephalosporins are unlikely to become widely used due to cost.^4^ Indeed, in assessing the potential benefit of any policy move towards widespread cephalosporin usage, improvements in efficacy will need to be weighed against the risk of promoting community-based third-generation cephalosporin resistance. In our setting in rural Kenya, increasing unregulated use of ceftriaxone in the community appears to be driving high levels of resistance to multiple antibiotics identified in bacteria isolated from community carriage (literally, where it is possible to identify the presence of microbes on skin/mucosal surfaces without direct evidence of them causing disease) and infection studies (unpublished). Data from Médecins Sans Frontières in Niger showed alarmingly high rates of carriage of highly resistant enteric bacteria in children with SAM in the community.^5^

Currently, WHO guidelines (which pre-date the Malawi study) recommend provision of co-trimoxazole as the ‘broad spectrum antibiotic’ for treatment of children with SAM in the community.^6^ Amoxicillin and co-trimoxazole have similar spectra of activity and are both widely used so may have similar problems of antibiotic resistance. Despite a widespread view that co-trimoxazole is less effective than amoxicillin in treating small intestinal bacterial overgrowth, in fact they have both been used with some success for this indication – though neither is as effective as other agents, like amoxicillin-clavulanic acid, metronidazole, or rifixamin.^7^,^8^ There are no published studies on the use of co-trimoxazole in severely acutely malnourished children treated in the community, whereas amoxicillin use is now supported by the Malawian trial. Amoxicillin reaches therapeutic plasma levels in acutely malnourished children when given orally, and is considered to be reasonably safe with few side-effects.^9^,^10^ Therefore, pending further studies or significant changes in the availability of oral third-generation cephalosporins, current evidence suggests that amoxicillin 80–90 mg/kg/day in two divided doses for 7 days is the most appropriate treatment. While other dosages and regimens have been quoted previously, this is now the one with the strongest evidence base.^4^ For children already taking daily co-trimoxazole prophylaxis because of HIV, this should be continued at the usual dose throughout their management, but an additional antibiotic (e.g. amoxicillin) should be given at presentation as well.

### Which antibiotic is most appropriate for inpatient care?

Where strong programmes of active early case-finding for acute malnutrition in the community are present, most malnourished children will be referred for care before the onset of complications, will be clinically well and have retained appetite. Such can be safely managed in outpatient care and do not require hospital admission.^11^,^12^ However, children who are severely unwell, fail an appetite test (unable to take or tolerate sufficient RUTF), or who have evidence of severe or systemic infection require at least a period of inpatient-based stabilisation prior to transfer for outpatient management of SAM. The mortality rates for such children are often very high, and invasive infection is often present. In a case series at our centre, a rural district hospital in Kenya, laboratory-proven bacteraemia occurred in 12% - the true proportion with bacteraemia is likely to have been much higher because the inherent sensitivity of blood cultures is low.^13^ These, and other data provide a clear rationale for the routine provision of antibiotics to children with SAM requiring inpatient care. WHO recommends ampicillin (parenterally for 2 days followed by enteral amoxicillin/ampicillin for a further 5) and gentamicin (parenterally for 7 days), though it is notable that the efficacy of this particular combination and schedule has never been tested in this population in a randomised controlled trial.

In hospital-based case-series, bacteraemia in severely acutely malnourished children is mostly caused by enteric pathogens (such as Salmonella species and *E. coli*), with a significant minority caused by Gram-positive organisms including Streptococci and *Staphylococcus aureus*.^13^–^15^ A number of studies also consider coagulase-negative Staphylococci (CONS), for example *Staphylococcus epidermidis*, which are frequently multiply antibiotic-resistant, as pathogens.^16^–^19^ CONS form part of the normal skin flora, and are frequent contaminants of blood cultures but rarely cause clinically significant bloodstream infection. Where they do, this usually occurs in the context of indwelling central venous catheters (owing to their propensity to form adherent biofilm on plastic) alongside immunosuppression. They are an important pathogen in premature neonates receiving total parenteral nutrition via a central venous catheter, for instance.^20^ Distinguishing a CONS-positive blood culture due to contamination to one from blood-stream infection is extremely difficult, and in sick malnourished children we consider that it is almost impossible at a patient-by-patient level.

Assessing whether CONS are pathogenic in malnourished children more generally requires that their presence in blood cultures be systematically associated with a clinical phenotype or outcome consistent with bacteraemia. Such assessments have not been reported. Therefore, whether or not CONS cause bacteraemia in malnourished children at first presentation remains controversial, and in the absence of further evidence it is difficult to judge whether empiric antibiotic regimens should be tailored to these highly resistant organisms. We currently regard CONS as contaminants in this context, partly because improved blood culture collection technique has dramatically reduced their detection (unpublished data).

Importantly, almost all studies reporting causative organisms for bacteraemia in severely malnourished children have come from sub-Saharan Africa (along with several from Jamaica). We were unable to find any similar studies from South and South East Asia despite evidence of significant differences in causative agents of bacteraemia more generally.^21^

While ampicillin and gentamicin have acceptable pharmacokinetic profiles in malnourished children (see Lazzerini & Tickell for a review on this and other aspects relating to antibiotics in SAM),^22^ there is a lack of studies addressing toxicity, including long-term follow up of renal function in children given gentamicin during SAM. Microbial resistance profiles vary widely in different studies. For instance, 85 to 96% of blood culture isolates from South Africa, the Gambia, and Kenya were sensitive to ampicillin/gentamicin combination therapy,^13^,^14^,^23^ but in a referral centre in Uganda less than a third were.^15^ Resistance is a problem in all geographical areas, but our understanding of the scale of the problem is hampered by a lack of recent high quality studies. Inferences based on studies from 10 or 20 years ago are likely to dramatically underestimate the prevalence of resistance to antibiotics that are now easily available in the community and widely utilised in many parts of the world. With this in mind, we consider there to be an urgent need for clinical trials assessing ampicillin/gentamicin against either parenteral ceftriaxone or oral ciprofloxacin, which has recently been shown to be bioavailable in SAM.^24^ These studies should include both clinical endpoints and measures of antimicrobial resistance. One on-going study in Bangladesh is assessing the use of ceftriaxone in severely malnourished children with pneumonia, but both arms are receiving parenteral ceftriaxone and are randomised to either hospital or home-based management. The study was due to finish in October 2011 but has not yet, to our knowledge, been reported (NCT00968370). Such an approach may be important, since in a rural Kenyan hospital, SAM was significantly associated with nosocomial bacteraemia (hazard ratio 2.52), emphasising the fact that shortening duration of hospital stay is important in these high-risk patients.^25^ To that end, the current WHO stipulation for 7 days parenteral gentamicin is impractical, and a comprehensive assessment of its efficacy is overdue,^6^ especially in the light of risks of nosocomial infection and potential for renal toxicity. Comparing outcomes of a shorter duration of gentamicin (e.g. 2 days) could be evaluated in a clinical trial of non-inferiority amongst children with complicated SAM but no specific signs of severe infection, bearing in mind a basic tenet of paediatric care that IV antibiotics should be switched to oral sooner rather than later in order to minimise discomfort and nosocomial infection risk from indwelling devices. There is also a strong case for considering other strategies, such as the use of amoxicillin-clavulanic acid from day one, which has a similar spectrum of activity and is likely to be more active (than amoxicillin) against small bowel overgrowth (see below in relation to metronidazole).

Finally, children with SAM and complications admitted to hospital continue to have a high risk of mortality following stabilisation.^26^,^27^ Our group is currently investigating whether provision of 6 months co-trimoxazole prophylaxis, analogous to that provided for HIV-infected children, impacts on mortality during 12 months in HIV-uninfected children aged 2–59 months admitted to hospital with SAM (NCT00934492). This study will report in early 2014.

### Should children with SAM be treated with metronidazole?

Metronidazole is an antibiotic with anti-anaerobic and some anti-protozoal activity. Its status in the management of SAM is currently unclear. The WHO Pocketbook states, ‘some experienced doctors routinely give metronidazole (7.5 mg/kg 8-hourly for 7 days) in addition to broad-spectrum antibiotics. However, the efficacy of this treatment has not been established by clinical trials.’^6^ While the lack of clinical trial data remains unchanged, there are sound reasons for considering its provision, especially where there is persistent diarrhoea.

Firstly, metronidazole is active against Giardia, which is widespread in some settings. In a small study from the Gambia, 45% of severely acutely malnourished children with chronic diarrhoea had giardiasis, along with 27% of malnourished children without diarrhoea.^28^ A recent study from Rwanda that used microscopy and molecular diagnostics to assess for the presence of *Giardia duodenalis* in stool from 583 children living in rural areas found that infection was significantly associated with malnutrition – amongst 53 children with SAM, 38% had giardiasis on microscopy, and 87% by a combination of microscopy and polymerase chain reaction (PCR) test. Diarrhoeal symptoms appeared to have no relationship with the presence of infection.^29^ High titres of anti-Giardial IgM were associated with severe malnutrition in rural Bangladesh, despite low incidence of microscopically confirmed disease.^30^,^31^ Giardiasis can be difficult to diagnose, especially in the context of malnutrition since their clinical features have significant overlap, and laboratory diagnosis is difficult, relatively insensitive, and frequently unavailable.

Secondly, metronidazole is active against small intestinal bacterial overgrowth. The duodenum and jejunum usually contain low numbers of resident commensal bacteria, but certain circumstances predispose to overgrowth with mixed pathologic and non-pathologic species.^32^ Achlorhydria, reduced gut motility and impaired absorption of sugars have been postulated to drive bacterial overgrowth in malnourished children, with a potential role for unhygienic food preparation practices. Overgrowth can be measured by non-invasive breath tests as well as via culture of small bowel aspirates. It is important to note that while there is a strong theoretical basis for bacterial overgrowth in malnutrition, there are few clinical data from which to assess likely prevalence, clinical importance, or response to treatment in different settings.^33^–^35^ In a trial of provision of rifamixin, an oral non-absorbable antibiotic previously shown to be effective in treating small intestinal overgrowth, to well-nourished children in Malawi, there was no impact on elevated intestinal permeability, which is a feature of a pattern of enteropathy frequently seen in severe malnutrition.^36^ Importantly, small intestinal overgrowth involves both aerobic and anaerobic species. While amoxicillin-clavulanic acid provides good anaerobic cover, amoxicillin on its own has limited efficacy against Bacteroides species, which are frequently present.^37^ Metronidazole alongside amoxicillin would be likely to provide more complete coverage.

Additionally, while anaerobic organisms have been considered rare causes of bacteraemia in children, recent data suggest that in immunosuppressed patients they may be more important pathogens than previously recognised.^38^ Anaerobic blood culture is not usually performed in children and no data exist on potential incidence of anaerobic bacteraemia in malnourished children. However, the fact that i) SAM is associated with increased gut permeability favouring translocation of gut flora, and ii) the devastating condition Noma (cancrum oris) appears to involve both severe undernutrition and invasive anaerobic infection,^39^ suggests that providing broad anti-anaerobic cover may be beneficial.

On the other hand, metronidazole has potential side effects in children that may complicate its use in SAM, including nausea, diarrhoea, taste disturbance and, crucially, anorexia.^40^ Furthermore, the only study specifically assessing pharmacokinetics of metronidazole in SAM recommended a reduced dose of a total of 12 mg/kg/day (rather than 22.5 or 30 mg/kg/day), because pharmacokinetic models suggested that higher doses could lead to accumulation of the drug. Metronidazole-induced hepatotoxicity may occur in children with SAM (Prof. Michael Golden, personal communication), potentially as a result of this effect.^41^ However, in other conditions where metronidazole is used in the context of undernutrition (e.g. paediatric inflammatory bowel disease), the higher dose is recommended as standard. The pharmacokinetic study was small, and the case definition of malnutrition was based on non-standard parameters. Reports of hepatotoxicity due to metronidazole are otherwise rare in the medical literature, though drug accumulation would be likely to increase the incidence of side-effects, which could be very harmful in the difficult early stages of managing SAM.^42^

Clear guidance on use of metronidazole cannot be given in the absence of clinical trial data, and a randomised-controlled trial can be justified and should be performed. Prior to this, we consider that reassessing pharmacokinetic properties of the drug should be a research priority because it may be that a lower dose would have a more favourable risk-benefit profile. In the meantime, there is some justification for considering routine use (this decision will be dictated by local guidelines/experience/expertise), though probably at the lower dose, and good evidence that it is worth trying in cases of persistent diarrhoea.

## ANTIPYRETICS

Paracetamol (also called acetaminophen) is frequently prescribed for children with and without SAM who have malaria and other febrile illnesses. Importantly, trials in children with malaria without SAM have shown either no overall benefit or delayed parasite clearance with paracetamol.^43^,^44^ In SAM, it is possible that the risk of hepatic damage due to paracetamol at normal doses is increased, especially during the stabilisation phase because of reduced metabolic function in the liver. This is supported by pharmacokinetic data: the elimination of paracetamol is considerably prolonged in severely malnourished children.^45^ For this reason, some expert groups have cautioned against the use of paracetamol in malnourished children.^46^,^47^ The WHO hospital guidelines advise that paracetamol should only be used when high fever is causing distress to the child.^6^ The risks and benefits of paracetamol in children with SAM need to be assessed in clinical trials, but in the meantime it seems prudent to avoid its use where possible in SAM. Clinicians should be guided by the child’s discomfort rather than the measured temperature. Tepid sponging is an alternative to paracetamol. It is important to remember that aspirin (also called acetylsalicylic acid) is contra-indicated in children because of the risk of Reye’s syndrome. There are no studies of the use of ibuprofen in children with SAM, however it is not recommended in children with dehydration due to potential impact on renal function and gastric bleeding.

## TUBERCULOSIS

Tuberculosis (TB) is caused by *Mycobacterium tuberculosis* (MTB), a slow-growing bacillus that is transmitted by respiratory aerosols. Children are usually infected with MTB by a household contact with pulmonary disease and a cough, productive of sputum. Following initial infection, MBT may be either contained (though not eradicated) by the child’s immune system, which results in latent TB infection (LTBI), or may progress to one of several clinical phenotypes. In adults, TB primarily causes respiratory disease, whilst in children, extrapulmonary disease syndromes such as lymphadenopathy, dramatically disseminated disease (miliary TB) or meningitis are common.^48^,^49^ The pattern of respiratory disease in children is different from that in adults, with less cavity formation, and sometimes TB infection is indolent, presenting with non-specific features such as weight loss and persistent fever.

The gold standard for diagnosing TB disease is culture of MTB, but obtaining suitable samples for culture in children is difficult. Children with pulmonary TB are often unable to produce sputum and infections tend to be pauci-bacillary (low numbers of bacilli), meaning that false negatives are common. This, coupled with the fact that MTB takes weeks to grow in culture, means that it is usually appropriate to start treatment on the basis of clinical features before receiving confirmation by culture.

Another set of methods for diagnosing TB infection are based around assessing for immune memory response to MTB, and include Tuberculin Skin Test (TST) and the Inteferon-gamma Release Assays (IGRAs), Quantiferon® and T-Spot TB®. These methods do not distinguish between latent and active TB, and they are of limited use in diagnosing active infection due to high false-negative rates.^50^ Other newer methods for TB diagnostics exist and may become more widely used in the next few years, but are mostly refinements to culture, and the difficulty of obtaining specimens in children remains.

WHO guidance indicates that TB diagnosis in children should be based on clinical features, supported by culture and microscopy, chest radiograph and TST/IGRA where available, and depending on the clinical syndrome. Children often catch TB from adults at home, and siblings may be infected as well. When a child is started on treatment, it is important to offer screening to all household contacts. Many countries have clinical scoring systems endorsed by national TB programmes to support diagnosis. Useful resources on TB may be found at the following:

- http://www.tbonline.info/guidelines/
- http://www.uphs.upenn.edu/bugdrug/antibiotic_manual/iautldtbkidsdxrx2010.pdf
- http://whqlibdoc.who.int/hq/2007/WHO_HTM_TB_2007.381_eng.pdf
- http://whqlibdoc.who.int/hq/2006/WHO_HTM_TB_2006.371_eng.pdf.

### What are the performance characteristics of clinical scoring systems for TB in SAM and/or HIV positive children?

A recent systematic review on clinical scoring systems for diagnosis of TB in children found a number of different systems, but no studies focussed on children with SAM, or compared validity of systems between SAM and well-nourished children.^51^ In part this is because acute malnutrition itself (especially if unresponsive to nutritional rehabilitation) is a criterion in many of the scoring systems. In cases of TB where acute malnutrition is a more prominent feature than cough, for example, it is also less likely that suitable specimens for gold-standard diagnosis will be available. Our approach (in line with WHO) is to consider the clinical response to nutritional rehabilitation and supportive treatment as being more important than a one-off assessment and score.^52^ For example, failure to gain weight despite consuming appropriate quantities of therapeutic feeds for a week or two (including F100 by nasogastric tube where appropriate), on-going fevers despite a course of antibiotics, or even persistent anorexia, should lead to strong consideration of a diagnosis of TB. In practice, empiric treatment will often be warranted, and treatment should never be delayed by waiting for culture results, especially in the more severe types of TB disease (e.g. suspected miliary TB or TB meningitis).

Another clinical feature that should be considered but is not mentioned in most scoring systems is the presence of rickets. We have found settings, specifically some urban areas in Kenya, in which the prevalence of active rickets among children with SAM is extremely high. While rickets in sub-Saharan Africa has usually been considered to result from calcium rather than vitamin D deficiency, vitamin D may be an issue where cultural practices promote low sunlight exposure for mothers and young children.^53^ Important recent studies have suggested that vitamin D deficiency or insufficiency may be associated with TB disease, and vitamin D has been shown to be important in anti-mycobacterial immunity.^54^–^56^ Further epidemiological studies are required to address this issue, but in the meantime it is essential that children with rickets be treated with both vitamin D and calcium regardless of setting. Clinicians should recognise the importance of carefully considering co-active TB disease in such children, as well as making a clinical assessment for rickets in those being started on treatment for TB.

### What are the performance characteristics of tuberculin skin test (TST) or interferon-gamma release assays (IGRA) in SAM?

As described above, these tests may be used to assist in the diagnosis of both latent and (to a lesser extent) active TB. For a clinician looking after children with SAM, it is detecting active disease that is by far the most important consideration and it is undoubtedly easier to assess for the presence of latent TB once children have nutritionally recovered.

SAM is associated with depressed delayed-type hypersensitivity responses, which would be expected to reduce the sensitivity of tests that rely on demonstrating cellular memory immune responses.^57^,^58^ IGRA tests are performed by measuring interferon-gamma (IFNγ) release by immune cells either i) without stimulation, ii) with a non-specific immune stimulant, and iii) with TB-derived proteins. The test can only be interpreted if the unstimulated IFNγ release is ‘low’ and the non-specifically stimulated release is ‘high’, otherwise the result is considered indeterminate. One important recent study found undernutrition to be associated with a high frequency of indeterminate results – presumably as a result of the general failure of cell-mediated immunity (and consequent failure of the internal positive control) as described.^59^ Although the TST does not have an equivalent ‘internal control’, the same effect is probably present, but would manifest as just a ‘negative’ result. This may be part of the reason that both TST and IGRA seem to perform poorly at predicting TB disease in areas where malnutrition is common. A systematic review showed that TST and both types of IGRA had substantially lower sensitivity for predicting active disease in low/middle-income countries than in high-income countries.^60^ In a recent study from Tanzania in a population with high levels of acute malnutrition (58% Moderate Acute Malnutrition (MAM) or SAM), TST and IGRA both had extremely poor sensitivity in picking up active TB (12% for predicting microbiologically confirmed TB and 18% for highly probable TB on the basis of presentation and clinical response to treatment).^61^ Importantly, in this study an indeterminate result was a risk factor for death. Another study from South Africa suggested that while malnutrition adversely impacted the sensitivity of TST (from 63% to 44%), it did not affect IGRA sensitivity.^62^ This study used an ELISPOT-based assay that enumerates IFNγ-producing cells rather than IFNγ levels overall, and might hypothetically be more appropriate where moderate cellular anergy, or ‘unresponsiveness’ exists.

Overall, the low sensitivity of both types of immune tests for active TB, and potential effect of malnutrition in exacerbating this, substantially limit their use in SAM. Most studies to date have grouped MAM and SAM together in reporting outcomes – we suggest that they should in future be separated out, which would provide more operationally useful data. For now, we consider that TB remains largely a clinical diagnosis amongst children with SAM.

### How is TB best managed in the context of SAM?

There are no data to suggest that drug treatment of TB should be altered in the context of SAM, but the importance of providing pyridoxine (vitamin B6) supplementation to children being treated with isoniazid should be highlighted. Isoniazid-induced polyneuropathy appears to be much less common in children than in adults at doses routinely recommended for treatment,^63^ although two small studies have shown that low vitamin B6 levels may occur before and during treatment, especially in the context of HIV.^64^,^65^ WHO recommends pyridoxine supplementation (throughout treatment with isoniazid) for severely acutely malnourished children at 5–10 mg/day in a single dose.^52^ RUTF contains pyridoxine at 0.6 mg per 100 g, which means that intakes will always be well below the recommended dose. On the other hand, it is highly unlikely that the small additional amount provided by RUTF (on top of supplementation) could cause adverse effects or ‘overdose’ if given together with the recommended dose of pyridoxine.^66^ Therefore all children with SAM should receive pyridoxine during treatment with isoniazid (probably for the whole treatment course although this is not specified and has not been tested).

When to start TB treatment is a decision that will be influenced by level of confidence in the diagnosis, clinical syndrome, and risk-benefit. There are no published data that suggests delaying treatment until completely/partially nutritionally rehabilitated, and such a strategy should not be employed. Children with TB are usually not infectious but their carers should be offered screening if visiting or staying on the ward. Isolation of a child thought to be infectious should be considered on a case-by-case basis.

## HIV

The HIV pandemic has changed the epidemiology, pathophysiology and mortality of SAM in many parts of sub-Saharan Africa and other areas of the world.^67^,^68^ Currently (as of early 2013), there are very few evidence-based recommendations for managing children with SAM and HIV infection any differently to children with SAM without HIV infection. However, drug toxicity, antimicrobial use, fungal infections and persistent diarrhoea are likely to require extra consideration amongst HIV-infected children with SAM.

### Should HIV testing be routine in all SAM programmes? Or otherwise at what level of HIV prevalence should it be mandated?

Worldwide, there is geographical overlap in the occurrence of HIV and malnutrition in children. In 2007, the WHO recommended that in high HIV prevalence areas, ‘Voluntary counselling and testing (VCT) should be available for children with severe acute malnutrition and for their mothers’ early in the management of acute malnutrition.^69^ This is at odds with the current strategy in many countries, which favour provider-initiated testing and counselling (PiTC) or diagnostic HIV testing and counselling (DTC) approaches at all health service contacts where this provides an immediate benefit for a child’s care (note that ‘TC’ refers specifically to the approach of testing then counselling for direct clinical care, rather than ‘CT’, which refers to counselling and testing in other contexts). This is certainly the case for children presenting with SAM, for whom the detection and treatment of HIV infection with co-trimoxazole prophylaxis and anti-retroviral treatment (ART) when required is life-saving. Furthermore, ‘high prevalence’ was not defined. WHO have defined a ‘generalised epidemic’ where HIV affects at least 1% of the general population,^70^ but we consider that there may still be substantial benefits to universal testing in populations with prevalence below this threshold. In Niger, where the estimated adult population prevalence of HIV was 0.8%, HIV infection was found in 9% of children who were admitted to hospital with SAM.^71^ A review of HIV in SAM in sub-Saharan Africa reported a range of prevalence of HIV amongst children with SAM in published studies from <5% to >50%.^72^ HIV was associated with almost a 3 times higher mortality during treatment of SAM.

There are very few published data on HIV prevalence in children with SAM in India, Pakistan, Bangladesh and elsewhere in Asia, but in general, population rates of HIV infection are far lower than in sub-Saharan Africa. The Indian Ministry of Health and Family Welfare guidelines in 2011 specify ‘Screening for HIV after counselling (only when suspected, based on history and clinical signs and symptoms)’. One published hospital-based series in urban Mumbai reported that 14% of unwell children admitted with SAM were HIV-infected, though this is unlikely to reflect most community settings.^73^

One potential benefit of universal HIV testing in the context of MAM or uncomplicated SAM is timely intervention as severely malnourished children starting ART have higher mortality and poorer subsequent growth than non-malnourished children. A study in Ethiopia identified the main risk factors for mortality amongst children starting ART to be severe wasting, severe immunosuppression defined by CD4 count, and anaemia.^74^

The existing data therefore suggest that HIV testing and counselling should be routinely offered for complicated SAM in all regions and uncomplicated SAM and MAM in regions of high HIV prevalence (>1% in pregnant women). With an absence of data on cost-effectiveness and clinical benefits of the provision of HIV testing in outpatient management of SAM in lower HIV prevalence areas, there is not an established threshold for HIV prevalence for offering universal testing. Until such studies are done, policy will depend on local funding priorities.

### Does SAM affect the reliability of rapid point-of-care tests for HIV?

Rapid HIV tests are based on detecting antibodies to the HIV virus. Infants under the age of 18 months may have antibodies from the HIV-infected mother, and therefore their diagnosis of HIV is confirmed by PCR tests that work by detecting viral nucleic acid in white blood cells. Often the PCR test is not available immediately. False positive and false negative tests may occur with both rapid tests and PCR tests. There have been no published studies comparing the reliability of point-of-care tests for HIV between children with SAM and well-nourished children. Such studies are needed.

### Does malnutrition lower the CD4 count?

Severe acute malnutrition is known to be associated with an increased mortality from infectious diseases, meaning that children with SAM are effectively immunodeficient. However the precise mechanisms underlying this relationship are unclear. One of the most consistently reported abnormalities is a reduced delayed-type hypersensitivity reaction in response to skin testing for tuberculin or candida antigens. This suggests that immune mechanisms involving some aspects of T cell function are affected by malnutrition (CD4 cells are a subset of T cells). Furthermore, several studies have reported that the thymus gland, where T cells mature and develop, is affected in pre- and post-natal malnutrition.^75^

Regarding the *number* of T cells, in a study in Zambia, the CD4 cell count amongst HIV-uninfected children admitted to hospital with SAM was the same as that in healthy controls.^76^ In the same study, it was found that amongst HIV-infected children with SAM, the CD4 counts were very low and continued to decline despite nutritional recovery. One study in Uganda has suggested that CD4 counts may differ in oedematous malnutrition,^77^ but this needs to be confirmed.

These findings suggest that although T cell function may be affected, malnutrition *per se* does not reduce the CD4 cell count and a low CD4 does not improve with re-feeding. However, it should be noted that malnutrition becomes more likely to occur together with a low CD4 count because of the increased metabolic requirements of uncontrolled HIV, anorexia, malabsorption and opportunistic infections, rather than necessarily being the cause of the low CD4 count. For example, in Dar es Salaam, wasting amongst children with HIV was most strongly associated with diarrhoea (odds ratio 22).^78^

### When should ART be started in treatment-naïve HIV+ve children presenting with SAM? Does it depend on whether complications are present?

Overall, amongst infants (SAM and non-SAM), early ART initiation is associated with a 4-fold reduction in mortality.^79^,^80^ Thus, many national guidelines recommend that all infants under 18 months in whom HIV infection is confirmed should start ART regardless of CD4 count or clinical stage. The key question relates to timing of ART initiation when HIV has been identified in the context of SAM.

There have been no clinical trials of the timing of ART initiation amongst HIV-infected children presenting with SAM. The arguments for starting ART soon (7 to 10 days) after transition to the rehabilitation phase of SAM treatment mostly relate to reversing a potentially fatal immunodeficient state and improving tissue repair and subsequent growth. In the case of SAM, there is a potentially beneficial effect of improved immunity to diseases that are otherwise difficult to treat such as cryptosporidium, and eventually a reduced risk of fatal sepsis. Improved growth may be due to beneficial effects of feeding where there is oesophageal candidiasis, benefits for absorption where there are parasitic infections, and a reduction of nutrient requirements by bringing HIV viral replication under control, reducing opportunistic infections and thus ultimately reducing inflammation.

It is clear that in profoundly immunodeficient individuals starting ART, the risk of mortality increases in the first 3 months of treatment, i.e. before immunological recovery. However, this risk is likely to be even higher if ART is not started until a child is no longer severely malnourished, and the period of risk may be extended. In the ARROW trial in Zimbabwe and Uganda, one in nine children with advanced HIV starting ART were hospitalised with SAM at a median time of one-month post ART initiation, mostly due to underlying infections.^81^

The arguments against starting ART early include potential metabolic stress, such as mitochondrial and liver toxicity. However, there are no data on the frequency and severity of these in children with SAM. There have been a small number of anecdotal reports of kwashiorkor occurring soon after ART initiation.^81^ This may be a presentation of immune reconstitution inflammatory syndrome (IRIS, see below). There are concerns that IRIS could be more prevalent in children with SAM. In Peru, IRIS in children was associated with more advanced HIV disease and at least one indicator of malnutrition,^82^ but this has not so far been more widely demonstrated and importantly, the risk of IRIS (which increases in the most severely immunodeficient individuals) may be exacerbated further by delaying ART.

Although there are no clinical trial data, observational evidence suggest improved outcomes from early initiation of ART. In Malawi, children receiving ART within 21 days of treatment in outpatient therapeutic feeding had a higher likelihood of nutritional recovery.^83^ It should be noted that in the interpretation of the findings of this retrospective study, it was not possible to control for the reasons why some children may have started ART later than others.

It is possible that a conclusive clinical trial will never be done as the general trend is towards earlier treatment with ART. It seems unwise to start ART during the stabilisation phase of complicated SAM because of potential metabolic disturbances and drug interactions (although there are little published data on these). Once appetite has returned following stabilisation of complicated SAM or in uncomplicated SAM managed on an outpatient basis, it seems sensible to start ART as soon as metabolic abnormalities are reasonably expected to have resolved, indicated by a return of good appetite. This is likely to differ between children, depending on the degree of abnormalities and response to therapeutic feeding, and time for parents or guardians to receive counselling on the use of ART. An average of 7 to 10 days seems appropriate. One exception is that unresponsive persistent diarrhoea may require ART to resolve (see below).

### Does SAM impact on anti-retroviral treatment choice?

There are no specific recommendations for choosing different ARV combinations in children with SAM. Importantly, there is a lack of data on pharmacokinetics in SAM. SAM is likely to be associated with changes in drug absorption due to reduced gastric acidity, villous atrophy in the small bowel, reduced plasma proteins to which some drugs bind, alterations in body composition affecting distribution, potential changes to the blood-brain barrier, and metabolic effects that may affect elimination.

Of the limited data available, use of a divided adult fixed dose combination tablet of stavudine, lamivudine and nevirapine commonly resulted in under-dosing of children in a study in Malawi. This was not associated with malnutrition, although no SAM cases were included.^84^ In other studies, anthropometric markers were associated with altered plasma nevirapine concentrations in children taking fixed dose combinations: in Malawi and Zambia stunting was associated with lower levels and wasting with higher levels.^85^ In India, lower nevirapine levels in stunted children were also seen.^86^

Several studies have reported a sustained growth response to ART, even though children mostly did not reach normal weight or height for age. Importantly, low baseline anthropometric values are associated with impairment of subsequent growth.^87^–^90^ A poor response to ART or clinical deterioration may be due to on-going infections or result from a syndrome caused by over-exuberant inflammatory responses occurring during immune reconstitution (Immune reconstitution inflammatory syndrome or IRIS). In either case, direct and indirect evidence of TB should be sought.

Long-term metabolic complications of ART have recently been reviewed by Musoke and Fergusson.^91^ They include lipodystrophy, dyslipidaemia, lactic acidosis, insulin resistance and osteopenia. However, there are almost no data specifically relating to SAM or the use of RUTF, and these are important areas for future study.

### Are there any special macro- or micro-nutrient requirements for children with SAM and HIV infection?

Recent systematic reviews have not identified evidence to support the development of guidelines on nutritional support for HIV infected children that differ from non-HIV infected children. A Cochrane review of nutritional interventions in HIV published in 2007 reported that there were no trials with mortality or disease progression endpoints. A small number of trials (eight) provided evidence that macronutrient supplementation increased energy and protein intake, but there was no effect on weight or CD4 count.^92^ Regarding micronutrient supplementation, another Cochrane review in 2010 included eight trials involving children. It concluded that vitamin A reduced all-cause mortality and zinc reduced diarrhoea morbidity, as they do in HIV-uninfected children.^93^ Evidence and recommendations for the use of vitamin A and zinc are given in more detail later in this brief.

### How should antibiotics be used amongst HIV-infected children with SAM?

HIV-infected children and HIV-exposed infants routinely receive daily co-trimoxazole prophylaxis in most HIV care programmes. Co-trimoxazole was originally given as prophylaxis against *Pneumocystis jiroveci* pneumonia (previously known as *Pneumocystis carinii* pneumonia, or PCP). However, trials amongst African children and other populations demonstrated that co-trimoxazole prophylaxis results in a large reduction (almost 50%) in all-cause mortality almost entirely unrelated to PCP incidence. Surprisingly, this occurred despite high prevalence of resistance to co-trimoxazole in most pathogens.^94^ Co-trimoxazole prophylaxis is also highly effective in preventing malaria, even in areas with a high prevalence of drug resistance, suggesting that co-trimoxazole may have a positive effect on host immunity to infection or providing unfavourable conditions to microorganisms attempting to establish an infection, rather than just a simple antibiotic/antimalarial effect. Where HIV is newly identified in the context of SAM, co-trimoxazole prophylaxis should be started immediately. The usual dose is 120 mg/day for infants under 6 months and 240 mg/day for children of 6 months to 5 years in three divided doses.

There have been no trials of alternative antibiotics for the treatment of infections in children with SAM and HIV. However, because pathogens occurring in children who are already taking co-trimoxazole are likely to be resistant to it, it is recommended that for routine therapeutic administration in uncomplicated SAM, amoxicillin be used rather than co-trimoxazole. For complicated SAM, ampicillin and gentamicin should be used as usual. In both cases, the child’s usual co-trimoxazole prophylaxis should be continued. Because the genes encoding resistance to different classes of antibiotics are often transferred between enteric Gram negative organisms together, the need for second and third line antibiotics, or specific investigation for urinary tract infection and other infections should be considered early in children who are not improving from a serious infection or who have persistent fever.

### How should HIV-associated diarrhoea be managed?

Recommendations for acute watery diarrhoea do not differ in HIV-infected and uninfected children with SAM (see below). Chronic diarrhoea is more likely to be present in children with HIV infection and this may be due to an increased prevalence of intestinal parasites and intestinal damage from HIV itself. As outlined above, it does appear that the routine use of metronidazole may be justified in this group. However, HIV infection is also associated with pathogens that are not easily treated. Studies in Zambia, Uganda, Thailand and Nepal have identified cryptosporidium and microsporidia to be common in both HIV-infected and uninfected children with SAM and prolonged diarrhoea.^95^–^98^ In a trial of the anti-parasitic drug nitazoxinide, there was some benefit in HIV-uninfected children with cryptosporidium, but none in HIV-infected children.^99^ In this context, there is evidence that ART, especially regimens including a protease inhibitor, may restore immunity to parasites including cryptosporidium and microsporidia with a clear clinical response.^100^–^102^ Cytomegalovirus infection also causes protracted diarrhoea in other contexts of immunodeficiency but has not been investigated with respect to SAM and HIV.

With regards to feeding; gut wall damage, villous atrophy and loss of brush border enzymes warrant consideration of alternative formulations of enteral feeds. Lactose intolerance affected a quarter of children with SAM with diarrhoea studied in Mulago, Uganda.^103^ Malabsorption of other disaccharides and monosaccharides such as glucose are also likely to occur. Fermented milk products have a lower lactose concentration and are likely to be cheaper than soy-based or elemental feeds. A comparison of a traditional diet with yoghurt versus soy feeds in Pakistan reported that diarrhoea improved in most children, but nutritional recovery was better with the soy formula.^104^ Conversely, in a small study in Jamaica, weight gain in children given a soy protein formula was lower than with cow’s milk-based feeds.^105^ However, this is likely to have been due to a lower zinc concentration and higher phytate (an anti-nutrient) in the soy-based feeds. Cost is often a limiting factor in consideration of alternative feeds, such as soy-based formulae.

Studies in the 1980s in Mexico and Bolivia have examined chicken-based feeds in malnourished children with protracted diarrhoea, which appeared to have similar outcomes to soy-based or hydrolyzed lactalbumin-based formulae.^106^,^107^ More recently, a trial in Zambia compared an infant elemental diet (Neocate®) to skim-milk and soy-milk (but not F100, unfortunately) in children with SAM with persistent diarrhoea.^108^ Although diarrhoea frequency was similar between groups, weight gain was greater in those treated with the elemental feed compared to the standard nutritional formulae. However, the cost of elemental feeds prevents their widespread use. Further research is needed to determine if local or less expensive formulations can be used, and if novel approaches such as pancreatic enzyme supplementation may be useful. Early transition to RUTF may be effective, but no clinical trials have addressed this.

In summary, in children with HIV-associated diarrhoea in SAM, acute watery diarrhoea should be managed as per non-HIV infected children. For children with persistent diarrhoea, routine use of metronidazole (discussed in detail above) and early ART initiation may be beneficial. Early transition to RUTF and the use of fermented milk or soy-based formulae may be of some value.

### Does HIV cause anorexia? How should this be managed?

HIV infection causes metabolic disturbances that result in anorexia. These may be compounded by gastrointestinal infections, side effects of drugs and swallowing difficulty. The medical management approach is to identify and treat opportunistic infections, and start ART at the appropriate time (discussed above). Nutritional management aims to follow the normal protocol for SAM, but a nasogastric tube is more likely to be needed. After that, feeds may need to be smaller and more frequent.

Swallowing difficulty may occur due to oesophageal candidiasis. This should be treated with oral fluconazole rather than with nystatin, which is effective only for oral rather than oesophageal candidiasis.^109^ Other causes of a sore mouth or swallowing difficulty include herpes simplex infection and aphthous ulcers.

### What is the guidance on exclusive breastfeeding for mothers with HIV?

Current WHO guidelines (as of 2012) enable HIV-infected mothers to exclusively breastfeed for 6 months and continue during complementary feeding at least until 12 months with very little risk of HIV transmission. Recent landmark trials showed clearly that ART given either to breastfeeding mothers, or as prophylaxis given to infants until 6 weeks after breastfeeding is discontinued, are associated with very low risks of HIV transmission.^110^–^113^

Trials in India, Zambia and elsewhere have confirmed that formula fed infants of HIV-infected mothers have higher mortality than those who are breastfed in the context of a Prevention of Mother to Child Transmission (PMTCT) programme.^114^,^115^ Formula feeding or other forms of replacement feeding cannot therefore be recommended for poor populations in such settings. As in developed countries, there may be some better-off individuals in developing countries with access to clean water and other resources for whom replacement feeding does not carry a significantly greater risk,^116^ but this is not the norm.

**Additional resources:** The African Network for Care of Children Affected by HIV/AIDS (ANECCA) handbook is available for download at: http://www.anecca.org/.

## MALARIA

Malaria is a common febrile illness in many tropical areas. The prevalence of malaria amongst children with SAM depends on the local transmission intensity of malaria, and may range from zero to the 40% reported in Mozambique in 2001.^117^ Important clinical features include fever, hypoglycaemia, anaemia, metabolic acidosis giving rise to deep acidotic breathing, seizures and impaired consciousness (cerebral malaria).

Hospital and community-based studies have conflictingly reported that acute or chronic malnutrition is either protective for malaria or a risk factor. Detailed discussion of this is beyond the scope of this brief, which focuses on management of malaria in the context of SAM. It is clear that acute malnutrition is an independent risk factor for mortality in children admitted to hospital with severe malaria.^118^,^119^

### Does the sensitivity or specificity of malaria diagnostic tests differ in children with and without SAM?

The clinical diagnosis of severe malaria is unreliable because the signs and symptoms of malaria overlap with other common febrile illnesses such as pneumonia, meningitis and sepsis. Current guidelines do not recommend blanket treatment with antimalarials of children with SAM. Diagnosis is by microscopic blood slide examination or rapid diagnostic tests (RDT) that detect parasite antigens. The sensitivity of both types of test increases with increasing blood parasite density. Microscopy may detect malaria parasite densities as low as 5 to 10 parasites/μl of blood, but this is highly dependent on the skill and experience of the technologist, the care taken in preparing the slide and examining sufficient high power fields. In practice, false positives are common.^120^,^121^ In general, *Plasmodium falciparum* histidine-rich-protein 2 (PfHRP_2_) based RDTs are more sensitive than lactate dehydrogenase (LDH) based RDTs. The WHO recommendation for RDT procurement requires a minimum detection score of 50% at a *P. falciparum* parasite count of 200 parasites/μl.

A significant risk of presumptively treating a febrile child for malaria without a malaria test result (or worse, with a false positive result), is that the treatment for the true cause of the illness may have been omitted.^122^,^123^ On the other hand, in high transmission areas, it should be recognised that children may have coincidental parasitaemia and so there may still be another cause of illness that also needs to be treated.^124^

Extensive evaluations of the sensitivity and specificity of RDTs, their effectiveness in various health care settings have been undertaken,^125^,^126^ but none has specifically tested their diagnostic performance in relation to SAM.

### Does the management of severe or non-severe malaria differ in a child with SAM from that in a child without SAM?

Oral treatment of falciparum malaria with artemisinin combination drugs is recommended in most regions, whilst intravenous quinine or artesunate are recommended for severe malaria. Artemisinin-based drugs act very rapidly and have few, if any, side effects. A small number of cases of genuine resistance have been reported on the Thai-Cambodian border and in Myanmar, although reports of prolonged parasite clearance time elsewhere suggest that resistance may ultimately develop. No studies have compared treatment outcomes between children with and without SAM using these drugs. Very few studies of the pharmacokinetics, toxicity or efficacy of antimalarials have included children with SAM.

One study in Uganda, examined the efficacy of two artemisinin combination treatments, dihydroartemisinin-piperaquine (DP) and artemether-lumefantrine (AL), by nutritional status. Both drugs cleared parasites in >99% of children aged 4 to 12 months by day 3 across all nutritional status categories studied, but no children with SAM were included.^127^

Quinine is important to consider because it is commonly the only drug available for severe malaria and has a narrow therapeutic index (a small difference between the therapeutic and toxic dose). Three studies relating to SAM have been published. In six Nigerian children with kwashiorkor, oral quinine had slower absorption, a lower maximum concentration and slower elimination than in well-nourished children, but no specific alterations of dosing were recommended.^128^ In Niger, amongst twenty acutely malnourished children, (defined as having at least two of weight-for-age, height-for-age or weight-for-height <−2 z-score), with and without cerebral malaria, intravenous quinine had a reduced volume of distribution and total plasma clearance compared to well-nourished children, but pharmacokinetic profiles were in the range of effective and non-toxic for both, suggesting no dosage modification is needed.^129^ Different findings were reported amongst eight ‘globally malnourished’ Gabonese children defined by the ratio of their MUAC to head circumference, using intramuscular quinine.^130^ The volume of distribution and plasma concentrations of unbound quinine did not differ between children with acute malnutrition and well-nourished children. However, drug clearance was significantly faster and concentrations at 12 hours were lower in malnourished children compared with control subjects. A more frequent dosing schedule (8 hourly) was recommended by the authors for malnourished children.

Malaria is associated with hypoglycaemia both in itself and indirectly due to the fact that intravenous quinine can cause hyperinsulinaemia. Current advice in SAM is to maintain blood glucose at 3 mmol/L or above, or where blood sugar cannot be measured, to treat if symptomatic, and prevent with regular feeds if non-symptomatic. Intravenous quinine should be infused in a 5% glucose solution.^6^ Quinine is also associated with other important toxicities including hearing loss and cardiac effects. Overall, no specific recommendation can be made regarding the use of quinine amongst children with SAM from the pharmacokinetic studies described above. However, it should be recognised that parenteral artesunate is now recommended by WHO for severe falciparum malaria in children. Artesunate is associated with faster parasite clearance and reduced mortality, and has none of the toxicity concerns of quinine and should be the treatment of choice in children with SAM and severe malaria.^131^

Treatment of malaria with sulfadoxine-pyrimethamine (SP) amongst Rwandan refugees in Zaire (now Democratic Republic of Congo) suggested that treatment failure was more common amongst malnourished children (kwashiorkor or weight-for-height <80% of the reference median). Malnourished children had higher initial parasite counts and slower parasite clearance.^132^ It is unclear whether these children were given folate supplementation, and its potential effect on treatment failure, given that the mechanism of action of SP is to block folate pathways within the parasite.

The efficacy of intermittent presumptive treatment in infancy with sulfadoxine-pyrimethamine (IPTi-SP) may be less effective in malnourished children. In one study in Ghana, the protective efficacy of IPTi-SP was halved in 'malnourished' children, who were defined as having one or more of weight-for-age, height-for-age or weight-for-height z-score less than −2.^133^ Further evidence of a diminished prophylactic effect of antimalarials in malnourished children (not SAM) comes from the study of DP and AL described above. Amongst those who had received DP (and were not on co-trimoxazole prophylaxis because of HIV), new episodes of parasitaemia were more frequent in malnourished than non-malnourished children.^127^

Artesunate is more effective and safer than quinine, and should be the treatment for severe malaria in SAM.^134^ Artemether-lumefantrine is safe and highly effective in uncomplicated childhood malaria. Both are recommended in the WHO guidelines for treatment of malaria (2010), which supersede the advice given in the 2005 WHO Pocketbook.^135^ Note that neither artemether nor lumefantrine act through folate pathways and thus are not affected by concurrent folate administration.

The management of dehydration, metabolic acidosis (giving rise to deep, laboured breathing) and shock has not been specifically studied in children with SAM and malaria. Current WHO recommendations are to manage fluids as described in the section on diarrhoea. Severe anaemia is a common feature of severe malaria, especially in younger children. There have been no clinical trials testing the threshold for blood transfusion amongst children with SAM and malaria. Again, specific guidance for transfusion in SAM is given in the WHO guidelines: transfuse children with a haemoglobin concentration <4 g/dl or at 4 to 6g/dl if there is respiratory distress on admission.^6^

## PNEUMONIA

A number of observational studies have indicated that children with SAM may have radiologic evidence of pneumonia without one or all of what clinicians would consider to be the typical clinical features of pneumonia (as defined by WHO and others).^136^,^137^ While this is extremely important in understanding the epidemiology of both conditions, at present it does not have a major impact on management.

As described in the antibiotic section above, all children with SAM should receive antibiotics: if they are well enough to be managed as outpatients (i.e. ‘uncomplicated’ with no indication of, for example, an underlying infection like pneumonia), they should receive oral broad-spectrum antibiotics (usually amoxicillin). If they require hospital admissions, they should be treated with intravenous (IV) ampicillin and gentamicin. This strategy is almost identical to WHO management of pneumonia in (non-malnourished) children – who if ‘very severe’ receive IV ampicillin and gentamicin for five days, followed by a switch to amoxicillin, or if non-severe are given oral broad-spectrum antibiotics at home.^6^ Therefore, amongst children with SAM who are hospitalised, it makes no difference to initial antibiotic treatment whether or not they have radiologic pneumonia (although treatment for very severe pneumonia, defined clinically, specifies IV antibiotics for five days whereas SAM treatment involves shorter duration ampicillin, see below). For children who are managed as outpatients, it is theoretically possible that some may have ‘occult’ (not clinically-evident) pneumonia – but they too would be treated the same as a child with pneumonia who was well enough to be managed at home.

Indeed, the observation that children with SAM may have radiologic abnormalities without other features of pneumonia may relate more to TB, which often causes chest X-ray changes (though probably rarely lobar consolidation) without marked distress, than to occult bacterial pneumonia. We recommend that children with SAM with an abnormal chest X-ray in the absence of clinical pneumonia (for example, without elevated respiratory rate or distress) should be carefully evaluated for TB with a follow-up convalescent X-ray if possible.

We have noted that aspiration of milk feeds can occur amongst children hospitalised with SAM, especially amongst those who have cerebral palsy or other swallowing difficulties. Clinicians should be aware of this complication and consider the safety of children’s swallow when prescribing F-75 or F-100. A high index of suspicion should be maintained for aspiration (which may be silent) in cases of sudden deterioration especially when associated with feeding. Aspiration pneumonia may require broader spectrum antibiotic cover than for community (or hospital) acquired pneumonia, and we typically use ceftriaxone and metronidazole. We recommend this in preference to chloramphenicol, which is suggested in the 2005 WHO Pocketbook, because of i) slower clearance and potentially increased toxicity of chloramphenicol in SAM because of reduce hepatic conjugation and ii) the need for both broad-spectrum and anaerobic cover.

## ACUTE WATERY DIARRHOEA

### How should nutritional status be assessed in acute watery diarrhoea?

Acute watery diarrhoea (AWD) refers to the presence of three or more watery or abnormally loose stools in the preceding 24 hours. In the initial assessment of a child with AWD, two key questions that have a direct bearing on the child’s management are i) is this child dehydrated, and ii) is this child malnourished? Assessing nutritional status in the context of diarrhoea is complicated by the fact that SAM and severe dehydration share some clinical features, and that dehydration directly alters anthropometric indices, such as weight, weight-for-height/length and mid-upper arm circumference (MUAC). Watery diarrhoea is extremely common in children with SAM at presentation,^138^ and is often accompanied by some degree of dehydration.^139^,^140^ Determining which children with diarrhoea are malnourished is important because the initial management is different. The different anthropometric criteria for assessing SAM are impacted on by dehydration to different degrees:

**Weight-for-height/length (WHZ/WLZ) z-scores:** Children who have diarrhoea and are dehydrated weigh less than normal on account of their fluid deficit. In a small study of children with gastroenteritis in Rwanda, the median percentage weight gain from presentation to discharge was 4.8%, and almost a third of children gained more than 10%.^141^ In a study from Bangladesh amongst children initially categorised as severely malnourished with cholera, the average weight gain during the first 24 hours of hospital treatment was 11%.^142^ Such substantial weight gains in groups of children with diarrhoeal illnesses not pre-selected on the basis of clinical dehydration indicate that weight loss is very common in AWD, and the use of anthropometric cut-offs that depend on weight is therefore likely to be problematic.

**MUAC:** Because MUAC reflects a composite of fat and lean tissue bulk in the arm, it too may be affected by dehydration, which leads to loss of lean tissue water. In rural Kenya, children admitted to hospital with any features of dehydration (80% of whom had diarrhoea) had a mean increase in MUAC of 1.0mm in the first 48 hours following admission, and a 2.9% increase in body weight; equivalent to an increase in MUAC of 0.4 mm per 1% weight gained. This meant that 19% of children initially diagnosed with SAM on the basis of MUAC had a ‘non-severe’ MUAC after fluid resuscitation. The misclassification rate was similar to that using WHZ score; 21% of children diagnosed as SAM on the basis of WHZ score had a ‘non-severe’ score after resuscitation.^143^ However, overall, MUAC performed better because it misclassified fewer cases of MAM in the presence of dehydration.

Assessing nutritional status in children with diarrhoea in whom there is some evidence of dehydration is therefore extremely difficult, and it may be that even with the best measure, 1 in 5 children will be misclassified as SAM due to dehydration alone. At present there is insufficient evidence to suggest one anthropometric assessment over another. Most studies have been performed in hospital environments and focussed on a more severe spectrum of disease. Information to inform practice in non-hospital settings is lacking. However, it is important to recognise that the evidence underpinning almost all strategies for case-management of malnourished children have used a case-definition based on admission anthropometry, and not on ‘rehydration-corrected’ measures. Therefore while re-checking anthropometry in the first few days after admission or enrolment to a nutritional programme seems like a sensible policy for children who had diarrhoea, it may be unwise to consider those whose anthropometry recovers rapidly as ‘misclassifications’ and routinely discharge them from nutritional care. An example of misclassification is given in Box 1. Diarrhoea is so common in acutely malnourished children that this problem of misclassification has usually not been controlled for in studies evaluating prognosis or treatment of children with SAM. Work addressing this issue by assessing outcomes in misclassified children, or trialling rapid transfer to supplementary feeding (based on follow-up anthropometry), would be useful, though may not be a public-health priority.

### How should hydration status be assessed?

Assessing hydration status in severely malnourished children is extremely difficult, because all of the WHO-defined features of severe dehydration (lethargy/unconsciousness, sunken eyes, delayed skin pinch, drinking poorly) may also be features of SAM.^6^ We consider clinical signs in general to be of limited use in assessing hydration in children with SAM, although a careful clinical assessment is essential to pick up those with dehydration and signs of shock (see below). At initial presentation or referral, taking a careful history of intake and losses is important, and parents or carers may be helpful in describing any acute (short-term) changes, for example recent sunken eyes. In those children with a history suggestive of dehydration (e.g. AWD), it is reasonable to assume the presence of at least some dehydration and provide cautious oral rehydration. It is crucial to monitor children carefully during this time, since the response to fluids may provide the best index of whether dehydration is/was present initially. Children who deteriorate clinically when given rehydration fluid should be switched to maintenance intake while they are carefully reassessed. The ‘reassessment’ is critical here, and shifting back onto maintenance fluids should never be considered an end in itself, but part of a process and a strategy for trying to determine the optimal fluid management for each child. For children in inpatient care, looking at trends or acute changes in weight (on a suitable scale, and always using the same one) is a particularly helpful pointer for the new development of dehydration or, indeed, over-hydration.

### When are IV fluids required in children with SAM with AWD?

In assessing the need for IV fluids, it is important to be able to clearly distinguish the presence of dehydration from the presence of shock. In adults, approximately 1/3 of total body water is extracellular, and 2/3 is intracellular. Of the extracellular fluid, approximately 1/5 is intravascular (i.e. plasma) and 4/5 is interstitial. If an adult donates 1 litre of blood, their intravascular volume decreases, but within an hour or two it will have returned to normal because physical, chemical and some hormonal signals trigger replenishment from other fluid compartments. The adult would not have been ‘dehydrated’, because dehydration implies a reduction in total body water and 1 litre is trivial (for adults) in that context, but they would have been briefly intravascularly depleted. If children become dehydrated due to high losses and/or inadequate fluid intake, intravascular volume is maintained in the face of falling total body water, as fluid is shifted from both the intracellular and extracellular spaces. Eventually, they may become so dehydrated that the intravascular volume cannot be maintained, and features of shock will develop.

The normal physiologic response to intravascular volume depletion is to increase heart rate and decrease peripheral perfusion in order to preserve blood pressure and to optimise flow to vital organs. This is sometimes termed compensated shock. Eventually these compensation mechanisms break down (for example, because the heart is pumping so fast it can’t be properly refilled between beats), and shock becomes ‘decompensated’ with a falling blood pressure. The quickest way to deliver fluids into the intravascular compartment is intravenously, and in decompensated shock (i.e. with low blood pressure) this route is recommended.^6^ However, in compensated shock or dehydration without shock, the physiological differences between IV and enteral provision of fluid are less obvious. In such cases, when delivering fluid to children intravenously, the instilled fluid and electrolytes are transported around the body as part of the bloodstream, and leave the intravascular space by diffusion/osmosis into dehydrated tissues. When delivering fluid enterally, exactly the same process occurs. Fluid is absorbed across the gut epithelial surface and into the bloodstream, transported around the body and delivered to dehydrated tissues. Therefore the benefit to IV administration is the speed with which the fluid enters the intravascular space, but the disadvantage is that where there is no (or minimal) intravascular depletion, IV administration risks intravascular overload, which strains the heart. On the other hand, absorption from the gut is slower and subject to some homeostatic control, and is (probably) lower risk for inducing intravascular fluid overload. Consequently the WHO recommends oral/nasogastric (NG) rehydration unless there is decompensated shock (e.g. signs of shock plus coma or lethargy, potentially indicating impaired cerebral perfusion).^6^

### What type of fluid and rate are recommended for oral or IV use?

Children with SAM have low total body potassium and high total body sodium (mostly intracellular), and consequently rehydration with normal/high level sodium solutions has been regarded as dangerous.^144^–^146^ ReSoMal (Rehydration Solution for Malnutrition) has been developed for use in SAM, and has lower sodium and higher potassium, magnesium, zinc and copper concentrations than standard oral rehydration solution (ORS).

The evidence base around effectiveness of different oral rehydration solutions for use in SAM with acute diarrhoea was one of the topics assessed in a recent systematic review of treatment options in SAM.^147^ Three trials were identified that compared oral rehydration solutions with lower sodium concentrations to conventional ORS.^148^–^150^ All three studies pre-date a change in the formulation of standard ORS that reduced the sodium content and osmolarity. Table 1 shows the concentrations of sodium and potassium and the osmolarity of the lower sodium rehydration solutions used in each of the three trials; the old-formulation ‘original’ ORS (which was the comparator in all three trials), along with current ORS and ReSoMal. In the Alam *et al* (2000) trial, the low-sodium solution was essentially identical to current ORS, trialled against what is now considered an excessively high-sodium ORS formulation.^150^ It therefore has no bearing on which of the current options available are most appropriate. Dutta *et al* (2001) compared an option that is intermediate between current ORS and ReSoMal, to the high-sodium ORS, and its results similarly lack clear relevance to the assessment of which current formulation is best in SAM.^149^ Only Alam *et al* (2003) assessed the currently available low-sodium ORS option.^148^ It is important to note that although ReSoMal features in WHO and most national guidelines, and is provided to thousands of malnourished children around the world every day, the only trial testing it directly had a sample size of 130.

**Table 1:** Concentrations of sodium and potassium and the osmolarity of different oral rehydration solutions

	Alam (2000)^150^	Dutta (2001)^149^	Alam (2003)^148^	Original ORS	Current ORS	ReSoMal
Sodium (mmol/l)	75	60	45	90	75	45
Potassium (mmol/l)	20	20	40	20	20	40
Osmolarity (mOsm/l)	245	224	300	311	245	300
Sample size (SAM)	81	64	130	–	–	–

Alam (2000) and Dutta (2001) both showed that their lower-sodium concentration rehydration solutions were associated with reduced duration of diarrhoea and frequency of bouts. In the only study to assess ReSoMal against ORS (Alam, 2003), there was no difference in children rehydrated at 12 hours. Stool output, urine output, fluid intake, calorie intake from supplemented food, duration of diarrhoea and weight gain before discharge are reported as being ‘similar between groups’.^148^ The study reported the frequency of children who became ‘over-hydrated’, defined as ‘>5% weight gain after correction of dehydration at any time during the study period with any of the following signs: periorbital oedema/puffy face, increased heart rate (>160/m), or increased respiration (>60/min)’: Fewer children in the ReSoMal group than the ORS group became ‘overhydrated’. The difference was not significant (4.6% versus 12.3% p = 0.20), but the study was not powered to assess this outcome. Importantly, while ReSoMal was associated with faster correction of admission hypokalaemia, it was associated with hyponatraemia (29% versus 10% at 48 hours, p = 0.017), including one child who had a hyponatraemic seizure.^148^ However, in this study almost 30% of enrolled children had cholera, and stool frequencies (alongside, presumably, total stool volumes) were very high: mean stool frequency in the 24 hours preceding enrolment was 12.5 and 14 per 24 hours in the ReSoMal and ORS arms respectively. The child who had a hyponatraemic seizure did not have cholera but had a very high purging rate (18 g/kg/h during the first 24 hours, equivalent to 432 g/kg in total – almost half their body weight), and a ReSoMal intake of 528 ml/kg in the same period. ReSoMal is not recommended for children with SAM either with suspected cholera, or with ‘profuse’ watery diarrhoea, because of the need to keep up with stool sodium losses. Instead, WHO recommend standard ORS for children with SAM and profuse losses. One important difficulty in practically implementing this recommendation is uncertainty over what constitutes ‘profuse’, and guidance on this matter would be helpful from policymakers, as well as action to ensure that the different indications for ORS versus ReSoMal are properly visible and understood by clinicians on the ground. Furthermore, it is important to note that ReSoMal should only ever be used in inpatient settings. Rehydration formulae are designed for the treatment, rather than prevention, of dehydration, and where children with SAM need them, they also require admission.

Therefore, the very limited data on the use of ReSoMal provides no evidence of improved efficacy in rehydration. ReSoMal corrects low plasma potassium more quickly than ORS but was associated with a significant risk of prolonged hyponatraemia, although this occurred when it was used in a clinical situation in which it may have been contraindicated. Thus, we feel that the evidence base for using ReSoMal in preference to standard low-osmolarity ORS in malnourished children is extremely weak. We consider this to be one of the areas requiring large-scale operational and pragmatic clinical trial data comparing ReSoMal with standard low-osmolarity ORS plus additional potassium. Demonstrating equivalence could allow substantial simplification of guidelines that would be of real benefit to front-line health workers.

There are no trial data on which to base recommendations for rate of oral rehydration in children with SAM and operational factors may be important in deciding what is most appropriate in different settings. Our practice conforms to WHO guidance emphasising frequent clinical reassessment.

For children with SAM and features of shock, the only trial examining fluid choice found no significant differences between Ringers Lactate and Half-Strength Darrows/5% Dextrose, but extremely high mortality in both arms.^151^ No trials have been performed of different infusion rates or volumes. The investigators of this study have suggested that current WHO practice may be excessively cautious and that higher volumes and rates are required. More observational and, ideally, trial evidence is needed urgently. It is important to note that while children with kwashiorkor are not ‘dehydrated’ (in terms of reduced total body water), they may well be intravascularly depleted – emphasising the need for clinical features of dehydration and shock to be assessed separately.

Studies involving non-invasive physiological measurements may be helpful in defining the clinical features of shock in children with SAM. It is notable that the descriptions of ‘incipient’ and ‘established’ shock given in the 1999 WHO Management of severe malnutrition manual differ significantly from the more objective definitions of shock, including amongst children with SAM, in the 2005 WHO Pocketbook.

The recent high-profile FEAST trial, which assessed the use of bolus fluids in African children with features of shock versus maintenance IV fluids, specifically excluded both children with SAM and those with AWD.^152^ Although it therefore has no direct bearing on the management of children with either condition, the fact that the results were surprising to most commentators highlights the importance of performing clinical trials addressing difficult critical care issues where there are strongly held expert opinions.

### Are different antibiotics indicated in the management of children with SAM with AWD?

While diarrhoea is very common in SAM, it may also be severe, prolonged, difficult to manage, and unpredictable.^138^ It is our practice to admit children with SAM complicated by diarrhoea for inpatient management, including provision of IV antibiotics. Where admission is not possible or desirable, oral antibiotics should always be provided because of the mortality benefit afforded to children with SAM managed in the community (see ‘antibiotics’ section, above). Where cholera is suspected, antibiotic treatment speeds recovery amongst those who are moderately or severely unwell: a variety of antibiotic regimens may be employed, but most authorities (including WHO and Centre for Disease Control, USA) recommend erythromycin as first line treatment in children.^153^ In the case of bloody diarrhoea, a fluoroquinolone (or other drug, depending on local sensitivities) should be given to provide cover against *Shigella* species.

### Is zinc supplementation required in the management of children with SAM with AWD?

WHO recommends the provision of oral zinc supplementation to children with AWD at a dose of 10 mg/day for 10 days (if less than 6 months) or 20 mg/day for 10–14 days (if older than 6 months), though several of the trials on which this recommendation is based excluded severely malnourished children. The optimal dose of zinc for children with SAM is unclear – while a recommendation of 2–4 mg/kg/day is often quoted (including by WHO), the International Zinc Consultative Group recommends 10mg per day, and various dosing strategies have been used in trials.^154^,^155^ Importantly, a randomised controlled trial comparing different dosing strategies in Bangladesh found that higher-dose supplementation strategies (6.0 mg/kg/day compared to 1.5 mg/kg/day) were associated with excess mortality (risk ratio of 4.53).^156^

Children taking recommended intakes of therapeutic foods that comply with WHO specifications will always be ingesting 2–3 mg/kg/day of zinc (Table 2), and most children will be approaching or exceeding the 20 mg daily dose recommended for children with diarrhoea. For instance, a 6kg child on 130 ml/kg/day of F-75 will receive a total of 15.6 mg zinc per day. Notwithstanding the fact that zinc bioavailability can be altered by co-administration with other micronutrients, we do not consider that there is any evidence of benefit for supplementing children with SAM with additional zinc than that given during normal nutritional rehabilitation, and the potential harm associated with additional high dose strategies means that this should be actively discouraged.

**Table 2:** Zinc content of therapeutic foods

Product	Zinc content
F-75	2.0 mg zinc per 100 ml
F-100	2.3 mg zinc per 100 ml
RUTF	11–14 mg zinc per 100 g (equivalent to 545 ml F-100, so 2.0–2.6 mg/100 ml equivalent)

## SEPSIS

### What are the features of sepsis in SAM?

The terminology applied to severe infection is confusing and often inconsistent. Terms like ‘sepsis’, ‘septicaemia’, and ‘bacteraemia’ may all be applied to a child with a proven blood-stream infection, but in fact, indicate quite different things. A consensus conference of definition of terms and syndromes has gone some way to establishing a unified frame of reference for resource-rich settings, and while not all are appropriate or relevant to settings where SAM is common, they introduce important concepts. ‘Sepsis’ is defined as presence of the Systemic Inflammatory Response Syndrome (SIRS) with suspected or proven infection.^157^ Presence of SIRS is established on the basis of abnormalities in two of four criteria; temperature, heart rate, respiratory rate, and leukocyte count (one of which must be abnormal temperature or leukocyte count). ‘Severe sepsis’ indicates sepsis alongside organ dysfunction (which in malnourished children will commonly be respiratory or neurologic), and ‘septic shock’ indicates sepsis with cardiovascular organ dysfunction (which may be defined by the 2005 WHO Pocketbook criteria of (all of) cold hands with slow capillary refill time (longer than 3 seconds) and weak and fast pulse). ‘Bacteraemia’ indicates the presence of a positive blood culture, and ‘septicaemia’ is probably not a helpful term.

In that sepsis is a syndromic diagnosis, the clinical criteria will be the same regardless of the presence of SAM, with the only proviso that SAM is such an important risk factor for bacteraemia that invasive bacterial disease can almost always be reasonably suspected.^158^ Guidelines on the emergency care of SAM must fit in with established frames of reference for clinicians working on the front line in settings where SAM is common. This means that terminology should reflect standard practice as exemplified in ETAT (Emergency Triage and Treatment) guidelines, and idiosyncratic terminology for children with SAM should be avoided where possible. If the preceding terms are to be incorporated into later versions of national and international guidelines, we would suggest one important modification – to include bradycardia as well as tachycardia as a feature of SIRS, since in Kenya this was a consistently poor prognostic feature in children with SAM, and bradycardia is recognised in young infants as a feature of systemic infection. The same may well be true in SAM, where functional reserves are similarly limited.^13^

### What is the emergency care for septic shock in children with SAM?

All children with SAM and sepsis require admission to hospital, parenteral broad-spectrum antibiotic cover (see discussion on antibiotics, above), and careful clinical assessment – particularly to assess for features of septic shock. Beyond that, there is no robust clinical trial evidence on which to base treatment decisions. Supportive care should include keeping the child warm, oxygen (to maintain saturations above 90%, or empirically if saturation monitoring is not available), and careful monitoring of glucose. WHO recommends immediate provision of 10% dextrose either as oral bolus of 50 ml (25 ml for infants less than 6 months) or IV bolus of 5 ml/kg in children with SAM presenting with features of shock.^6^

Key management decisions in children with septic shock are how much fluid to give, how quickly to give it and what type to use. These decisions are extremely difficult in the context of SAM, and are controversial. Most authorities recommend fluid resuscitation at a slower rate than recommended for non-malnourished children, with relatively hypotonic solutions, and avoidance of the IV route where possible. The rationale for this approach is that clinical features consistent with volume depletion in SAM may be a consequence of a ‘reductively adapted’ cardiovascular state, with myocardial atrophy, low stroke volume, and a heart that is extremely sensitive to volume overload. On the other hand, it has also been suggested that the opposite may be the case, and that current guidelines are not sufficient to correct underlying volume deficits.^151^

In Uganda, mortality amongst children with SAM was found to be significantly associated with administration of IV infusions or blood transfusion, and these were likely to have been the more severely ill of the cohort studied. However, aligning practice to WHO recommendations on restricting these practices resulted in no overall reduction in mortality.^159^,^160^ Detailed haemodynamic studies using non-invasive monitoring (e.g. with echocardiogram) will provide important and useful information to move this debate forward. One such recent study in Egypt found that malnourished children had elevated troponin T (a marker of cardiac myocyte damage) and impaired left ventricular systolic function compared to controls, though it is unclear how many of them had infection or sepsis and the response to fluid administration was not studied.^161^

Pending further studies to provide evidence of benefit of another strategy compared to the current WHO guidelines, it seems reasonable to apply these as a default position. The guidelines focus on oral or nasogastric fluids where possible. When IV fluids are used, it is at 15ml/kg over one hour rather than a fast ‘bolus’.

The use of ‘maintenance’ IV fluids, the arm with the better outcome in the FEAST trial has not been studied amongst children with SAM. It is important to recognise that a single guideline cannot entirely address complex pathophysiology and variable contributions of different aetiological agents and degrees of metabolic or cardiovascular dysfunction in children with SAM. Thus, it cannot replace an experienced clinician and frequent reassessment. It should also be noted that the key aim of community-based or integrated management of acute malnutrition is early recognition and treatment of SAM and its associated infections in order to avoid severe complications.

## MEASLES

Measles is highly contagious and outbreaks can occur in populations where less than 10% of the population is susceptible.^162^ While overall global measles mortality is declining, large outbreaks in sub-Saharan Africa and slow implementation of optimal vaccination strategies in India mean that measles still accounted for an estimated 139,300 deaths in 2010.^163^,^164^

The epidemiological association between measles mortality and malnutrition, especially vitamin A deficiency, is well recognized.^1^ Recent work in animal models has begun to unravel potential mechanisms, with the discovery that vitamin A metabolites are fundamental in generation of Th1-type effector immune functions.^165^,^166^ The clinical and immunological correlates of vitamin A deficiency or supplementation are extremely complex and still under active investigation by a number of research groups. A comprehensive summary of the current state of research has been published.^167^

### How should children with SAM and measles be treated?

All children with measles who have SAM should be treated as severe complicated measles according to WHO guidelines, and require inpatient management.^6^ Alongside standard supportive care, the specific treatment for measles is vitamin A supplementation (VAS): 50,000IU (children <6 months), 100,000IU (6–11 months), 200,000IU (12 months or older) once per day for two days, and a third dose after 2 weeks (on day 15).^6^ Measles vaccination will be provided (see discussion below). Ocular or oral complications should be managed according to standard guidelines (tetracycline or chloramphenicol eye ointment/drops and atropine drops in case of corneal ulceration; regular salt-water mouthwashes alongside 0.25% gentian violet applied directly to painful mouth ulcers and systemic antibiotics (benzylpenicillin and metronidazole) if very severe).^6^

These recommendations are based on evidence that has been summarized in a recently updated Cochrane Review, which performed meta-analysis on three trials comparing two 200,000IU doses to a control (placebo or routine care without vitamin A) for treatment of measles severe enough to require hospitalisation.^168^ The risk ratio for mortality was 0.4 with VAS (95% CI 0.19, 0.87, p = 0.02), and the effect was especially pronounced in children under two years old. Of the studies included in the meta-analysis, Coutsoudis et al reported that no participants had SAM, and while Hussey et al reported that 38% of participants had WHZ scores below the 5th percentile (equivalent to z score −1.64), there was also no reported SAM.^169^,^170^ Only Barclay et al included some children with probable SAM: 25 (14%) had weight-for-age <60% (marasmic by Wellcome classification), but there were no children with kwashiorkor.^171^ On its own, this study failed to show a statistically significant reduction in mortality with VAS (p = 0.13), but reanalysis of the published data suggests that a positive effect of supplementation in well-nourished children may have been partially obscured by inclusion of children with SAM. Amongst the 25 marasmic children, more were given vitamin A than control (17 and 8, respectively); 4/17 (24%) children died in the VAS arm, and 3/8 (38%) in the placebo (p = 0.64). These 7 deaths accounted for 39% of total mortality in the trial. If the children with SAM are excluded from the analysis, the protective effect of VAS in non-malnourished children becomes much closer to being statistically significant (2.8% versus 10.7%, p = 0.066).^171^ Therefore, while there is good evidence for VAS in treatment of measles amongst well-nourished children, there is a paucity of evidence from clinical trials amongst children with SAM, and at least a suggestion that the beneficial effect may be less.

This is important, because the safety and efficacy of even a single dose of vitamin A in children with SAM has been called into question – especially in those with kwashiorkor, who were completely unrepresented in studies of VAS efficacy in measles. The WHO 2003 and 2005 guidelines recommended giving a single therapeutic dose of vitamin A (dosage as above) to all children with SAM on day 1, followed by daily low dose (as part of the micronutrient enrichment of therapeutic feeds – each sachet of RUTF contains in the order of 3000 IU retinol, for example).^2^,^6^ However, in a study from Senegal, a subgroup analysis of a study comparing single therapeutic dose VAS to a daily physiological dose found that children with oedematous malnutrition had higher mortality with the high-dose (200,000 IU vs 5000 IU/day) approach (odds ratio 0.21, p = 0.049).^172^ In an earlier study by the same group, daily low-dose VAS significantly reduced the risk of severe nosocomial diarrhoea in oedematous children compared to high dose.^173^ Some national guidelines (for example, Kenya, many from West Africa), now recommend withholding VAS on admission to hospital or enrolment to outpatient care in children who present with oedema unless there are clinical signs of Vitamin A deficiency or an active measles epidemic.^174^ The revisions to the WHO guidelines in 2012–2013 have acted on this evidence and the recommendation for routine provision of high-dose vitamin A have been withdrawn, except in cases where there is an ongoing measles outbreak or in the absence of the provision of therapeutic foods that comply with WHO specifications.

With the incidence of measles waning in most areas due to increased vaccination coverage, and with an epidemiological profile of intermittent outbreaks rather than endemic transmission, further trials on management of measles infection will be challenging to perform. In the absence of specific high-quality data on the effect of VAS in measles complicated by SAM, it is therefore probably appropriate to provide supplementation along the same lines as for well-nourished children.

Finally, while provision of broad-spectrum antibiotics should be routine in all children with SAM requiring admission, it is noteworthy that there is also evidence supporting antibiotic treatment for the specific management of measles to prevent pneumonia (i.e. not restricted to malnourished children): A recent high-quality randomized-controlled trial from Guinea-Bissau showed reduced pneumonia and/or conjunctivitis in children with measles randomised to 7 days of prophylactic co-trimoxazole compared to placebo, suggesting that broad-spectrum antibiotics should probably be routine for all children with measles who require hospital admission.^175^,^176^

### Should children with SAM receive measles vaccination during nutritional rehabilitation?

WHO guidelines^6^ suggest routine provision of measles vaccine (MV) to children with SAM in the circumstances outlined in Table 3: Where vaccination is recommended, it should be provided on admission, with a second dose on discharge. The stated rationale is that the admission dose is intended to ‘ameliorate the severity of incubating measles and partially protect from nosocomial measles’, and is not expected to provide a definitive and long-lived protective antibody response, whereas the dose administered after a period of nutritional rehabilitation is considered to be more likely to provide a protective antibody response.^174^,^177^ In children managed as outpatients, the initial dose may be omitted (since they are not being admitted) and a single vaccination should be given after one month’s nutritional rehabilitation. The evidence in favour of this approach appears to be weak. In a systematic review of interactions between nutritional status and vaccine responses in children, a number of studies that assessed response to measles vaccination during SAM were described.^178^ Although a few studies appeared to show reduced or delayed acquisition of protective antibody levels (titres), especially in the presence of kwashiorkor, the authors found that ‘…globally, [observational] studies did not show any association between malnutrition and the immune response to MV. Children with kwashiorkor, marasmus, or different degrees of [malnutrition] defined by weight-for-age all had high seroconversion rates to the vaccine.’^178^ Therefore the suggestion that delayed vaccination is more likely to result in protective titres is not supported by available evidence. Furthermore, we have been unable to find any evidence to support the utility of the admission dose in reducing severity of ‘incubating’ measles or preventing nosocomial measles in hospitalized children.

**Table 3:** WHO recommendations for measles immunisation

Age	WHO recommendation
<6 months	No recommendation for vaccination.
6–9 months	Vaccinate if not previously immunized.
>9 months	Vaccinate if not previously immunized or if immunized when younger than 9 months.
In all cases withhold vaccination if the child is shocked.	

**Figure 1:**
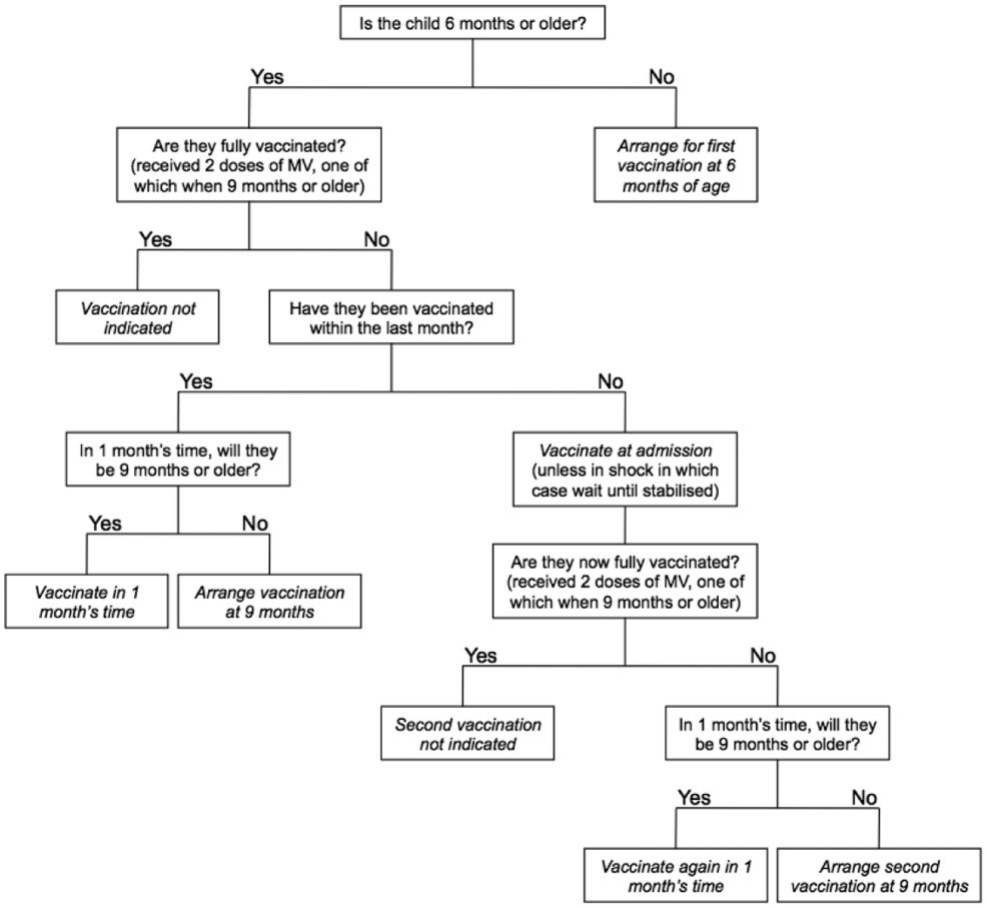
Measles vaccination scheme for children with SAM admitted as inpatients or managed in the community. This decision tree has been drawn by the authors, reflecting current WHO guidelines.^6^

That said, for the time being we consider this current approach to be broadly sensible. Reduced or highly variable efficacy of MV has been demonstrated in a variety of resource-poor settings where malnutrition is common, potentially partly due to programmatic factors.^179^–^181^ WHO recommends that all children should receive two doses of MV, and that the minimum duration between vaccinations is 1 month.^182^ At least one of the vaccinations should be given after 9 months, since efficacy (in terms of acquiring protective antibody levels) is greatest after this time point. WHO guidelines can be summarised as a decision tree (Figure 1).

In all cases, documentary evidence of previous vaccination is required – or the assumption should be that it has not been given, and the child should be vaccinated. For outpatients, there is no evidence that providing vaccination early jeopardises the development of protective antibody responses, and in our setting defaulting from care (and, for that matter, absconding from hospital) is a common problem that cautions against putting off interventions that could reasonably be done as soon as possible. Therefore exactly the same scheme could equally apply children with SAM managed as inpatients (where initial assessment should be made on admission to hospital) or as outpatients (where initial assessment should be made at enrolment). Malnourished children are often a socially marginalized and hard-to-reach group, who often (almost definitively) ‘slip through the net’ of public health systems. Where strong systems exist that could ensure delivery of a second MV dose, there may be arguments to increase the time gap between doses, but we believe that the giving a second dose during nutritional rehabilitation is a pragmatic and reasonable option.

Although accurate in terms of reflecting current WHO advice, the decision tree, above is quite complex; perhaps needlessly so. It could be streamlined by simply recommending provision of MV at admission (to hospital or outpatient care) with a second dose on completion of treatment regardless of age and vaccination status. Such an approach would mean than a few young children with SAM might end up receiving both doses before reaching 9 months of age, but it is important to note that while the justification for delaying measles vaccination to 9 months is that this provides optimal protective antibody levels, some have argued that it should be provided earlier. A recent review outlines these arguments, which are (partly) based on the fact that measles vaccination appears to improve survival in ways not only related to the prevention of measles.^183^ The authors conclude that ‘providing MV at 4.5 months of age … and again at 9 months of age, may significantly improve child survival.’ This is an extremely important debate even as measles incidence declines, and is an area to watch out for potential developments in the coming years.

## URINARY TRACT INFECTION

Urinary tract infection (UTI) is a common infection in childhood amongst children without SAM, affecting girls more often than boys. UTI may lead to renal scarring, acute renal infection leading to renal damage, and may act as a site of origin for systemic sepsis. Clinical features of UTI include fever, haematuria, strong smelling urine, increased frequency of urination, urinary incontinence, abdominal or back pain, and painful urination. In some cases, fever may be the only symptom. However, it is not known whether the febrile response to UTI in a child with SAM is altered. Signs on examination are usually absent but bladder infection (cystitis) may cause suprapubic tenderness and renal infection (pyelonephritis) may cause tenderness in the flanks. Paediatric guidelines in the United States and Europe recommend that all children aged less than 24 months with a fever of 38°C or more, and no other obvious focus of infection, should be evaluated for UTI.

### How is UTI diagnosed?

The diagnosis of UTI in a child with symptoms or signs is ideally made by Gram stain and culture of a urine specimen collected by suprapubic aspiration or catheter inserted for the purpose of collecting the sample. A ‘clean catch’ is the next best type of specimen performed by catching urine mid-stream. All of these may be difficult to collect before antibiotics are given, especially when a child is dehydrated. Reliable urine culture facilities are not normally available in most settings where SAM is managed and diagnosis relies on microscopy (>10 leucocytes per high power field or Gram stain for bacteria) or urine dipstick testing. A recent review reported that positive dipstick tests for either leukocyte esterase or nitrites have sensitivity and specificity for bacteriologically confirmed bacteriuria of 88% and 79% respectively, compared to 88% and 92% for urine microscopy for leucocytes and 91% and 96% for microscopy with Gram stain to visualise bacteria.^184^ The performance of urine dipsticks has not been specifically tested amongst children with SAM who might differ from well-nourished children in their white cell responses to infection. Findings have been inconsistent on this issue. In one study in India of 57 children with SAM, all those with bacteriuria had leucocytes detected on microscopy.^185^ In Nigeria, two thirds of children with SAM and bacteria detected in the urine had less than 5 leucocytes per cubic millimetre.^186^

### How common is UTI in children with SAM?

UTI is more common in malnourished than non-malnourished children, and the risk of UTI increases with the severity of malnutrition.^185^ Most studies of children with SAM have been conducted amongst sick children treated in inpatient facilities at tertiary centres, and have reported a high prevalence of UTI, ranging from 11% in Nigeria;^186^ 17% in The Gambia;^15^ 23% to 42% in South Africa (highest in HIV infected children and in those with marasmic-kwashiorkor);^187^–^189^ 24% in Kenya;^190^ approximately 30% in India, Bangladesh, Turkey and Uganda;^77^,^185^,^191^ and 37% in Ethiopia.^192^ In these studies, specific clinical signs of UTI were infrequent. In Kenya, a positive dipstick test was associated with increased mortality.^190^ In autopsy studies, between 15% and 71% of children with SAM who died had evidence of renal infection: abscesses or pyelonephritis.^193^,^194^

### Which bacterial pathogens cause UTI in children with SAM?

The leading bacterial causes of UTI appear to be similar to those in well-nourished children with *E. coli* and *Klebsiella* species being commonest. *Proteus, Enterobacter* and *Pseudomonas* species are also reported. Several studies have reported a high prevalence of resistance of organisms causing UTI to commonly used antibiotics amongst children with SAM. The results are very likely to be biased by being performed at referral centres and by antibiotic use prior to sampling (not assessed by urinary antimicrobial assays in these studies), which would ‘filter’ positive results in favour of resistant bacteria. In Kenya, 93% and 43% of urinary isolates were resistant to co-trimoxazole and gentamicin respectively.^190^ In Turkey, 82% of *E. coli* isolates were resistant to co-trimoxazole, but all were sensitive to gentamicin.^191^ In Nigeria, 77% and 23% of urinary isolates were resistant to co-trimoxazole and gentamicin respectively.^186^ Importantly, only 23% and 27% were sensitive to co-amoxiclav and cefuroxime respectively. In The Gambia, sensitivity of urine and blood bacterial isolates were reported together.^14^ None of the *E. coli* isolates were sensitive to co-trimoxazole or ampicillin, all were sensitive to gentamicin, nitrofurantoin, ciprofloxacin, and 2nd and 3rd generation cephalosporins. There have been no studies of treatment outcomes of UTI in relation to *in-vitro* resistance in SAM. There are no published data on tuberculosis or fungal infections of the genitourinary tract in children with SAM, but these should be considered in persistent UTI and if there is known TB exposure.

### Should children with SAM be routinely screened for UTI and what treatment should be given?

There have been no randomised trials of diagnostic strategies or different treatments for UTI in children with SAM. Given the high prevalence of UTI amongst children with complicated SAM, and association with mortality, in our opinion it would seem prudent to test and treat all children admitted with complicated SAM using urine dipstick or microscopy. However, it should be recognised that the recommended routine broad spectrum antibiotics (ampicillin and gentamicin for 7 days) for complicated SAM would be expected to cover the commonest community-acquired organisms causing UTI, provided there was not a high local level of resistance to gentamicin. Thus, the need to routinely test at admission depends partly on the local resistance patterns (which are often not known), and partly on resources. The ability to culture urine provides the possibility of individually tailoring treatment (especially if sensitivity testing is possible), but is not usually available and may not reveal all of the pathogens involved.

If a febrile illness in a child with SAM does not improve after 48 hours of intravenous antibiotics, we recommend that urinary testing should be performed. Where a UTI has been identified by dipstick or microscopy at admission in an area where antimicrobial resistance to the first line agents is confirmed, or after 48 hours of first-line antibiotics, an antibiotic specifically targeted at Gram negative organisms should be added, such as a cephalosporin, ciprofloxacin, nitrofurantoin or nalidixic acid.

In an outpatient setting, children with SAM and a high temperature should be referred and admitted as having complicated SAM. There are no published studies of UTI amongst children meeting criteria for uncomplicated SAM. Thus, existing data do not suggest a need to test every child with uncomplicated SAM for UTI, however, we recommend routinely testing febrile children, in the same way one would routinely test for malaria (in endemic areas), including amongst outpatients. Where there is known to be resistance and urine testing suggests UTI, one of the antibiotics listed above should be added, or the child referred to inpatient care.

### What further investigations should ideally be performed in a child with SAM and UTI?

For children aged 6 to 60 months, no further investigations are recommended for a UTI that responds well to treatment. Children with recurrent or persistent UTI require referral for further investigation to identify anatomical abnormalities and sites of damage or persistent infection. Measurement of plasma urea or creatinine is essential to identify renal impairment.

Further investigations depend on resources, but should ideally include an abdominal ultrasound scan to identify structural abnormalities. Where resources are available, this may lead to a micturating cystourethrogram and/or a dimercaptosuccinic acid (DMSA) scan at a tertiary centre.

Given the high prevalence of UTI amongst children with SAM in the published literature and concerns over antimicrobial resistance, more widespread data using standardised microbiological methods are needed. In particular, studies are needed to establish the prevalence of UTI in outpatient care programmes and the response to treatment and subsequent renal function with the currently recommended routine first line antibiotics given to children with SAM. Assessment of the performance of urine dipsticks and microscopy against the gold standard of urine Gram stain and culture is also important. UTI appears to receive a low level of emphasis in the 1999 WHO guidelines.

## NOSOCOMIAL INFECTIONS

Because of their duration of hospitalisation, susceptibility to infection and exposure to other children with transmissible infections, children with SAM treated in inpatient facilities have a high incidence of nosocomial infection. In Tanzania, 49% of severely malnourished children developed a new infection whilst hospitalised, most commonly sepsis and UTI.^195^ Causative organisms matched those on towels, beds and sinks. In Australia, amongst aboriginal children managed for malnutrition, nosocomial infection occurred in 38%.^196^

In Kenya, nosocomial bacteraemia was more than twice as common in children with very low weight-for-age.^25^ In this study, nosocomial bacteraemia was associated with a case fatality of 53%. In another study in South Africa, malnutrition was also the leading risk factor for nosocomial infection.^197^ Interpretation of some of these studies is made difficult as signs of new infection may overlap with those of re-feeding diarrhoea, aspiration pneumonia and signs appearing because of immune reconstitution. In an MSF programme in Niger the carriage of extended-spectrum β-lactamase producing enteric bacteria increased from 31% to >90% amongst children being treated for SAM in a therapeutic feeding centre that used ceftriaxone.^5^

Overall, these studies point to a very strong need for a high level of infection control in inpatient facilities managing SAM, including hand washing for patients, parents and staff, laundry, and separating patients with potentially infectious illnesses (e.g. acute watery diarrhoea) from the general ward. Use of rectal thermometers should be discouraged. The policy for use of antibiotics likely to rapidly induce antimicrobial resistance of clinical importance, such as ceftriaxone, needs careful consideration. A significant advantage of outpatient care, and a corresponding reduction in the duration of inpatient care, is less exposure to nosocomial infections, and this is acknowledged in the 2013 revision of the WHO management of SAM manual.

## SOIL-TRANSMITTED HELMINTHS

The soil-transmitted helminths (STHs) are parasitic worms that cause intestinal infections. They comprise *Ascaris lumbricoides*, hookworm species (*Ancylostoma duodenale*, *Ancylostoma ceylanicum*, and *Necator americanus*), and whipworm (*Trichuris thichiura*). Infection with STHs is extremely common in many parts of the world. It may be asymptomatic, or may result in gastro-intestinal symptoms due to the presence of worms in the intestinal lumen, or a range of distal symptoms depending on the species responsible. All STHs may contribute to undernutrition by competing for nutrients in the bowel, sustaining chronic inflammation, and causing malabsorption. They are an important cause of chronic anaemia. Control strategies for STHs are based around improvement in sanitation and population-based mass administration of anti-helminthic drugs, which appear to have somewhat limited and variable efficacy on growth and have been discussed in detail elsewhere.^198^–^200^

### Which drugs should be used to treat soil-transmitted helminths in children with SAM and when should they be given?

In terms of the specific management of helminth infection in children with SAM, WHO recommends treatment with mebendazole 100mg twice a day for three days where there is evidence of infestation, or routine provision of mebendazole in the absence of clinical/laboratory features in areas where infestation is very prevalent. Provision may be deferred until stabilisation where there are concerns the child may vomit the drug.^6^ Such an approach is based on very limited evidence, and may be due for an update: a systematic review of the efficacy of drugs used for single-dose mass drug administration found that although both albendazole and mebendazole were highly efficacious against *Ascaris*, they lacked similar efficacy against hookworm and whipworm, with albendazole slightly outperforming mebendazole.^201^ A randomized-controlled trial in older children and adults in China found that although a three-day regimen of either drugs was more effective than a single dose, albendazole was significantly more effective than mebendazole against hookworm, with both drugs performing similarly very well against *Ascaris* and similarly poorly against whipworm.^202^ Longer-duration treatment with albendazole is particularly attractive as it is also an effective treatment for Giardiasis.^203^ To our knowledge there are no major differences in resistance, side effects, or cost between albendazole and mebendazole, and it may be that a switch away from using mebendazole is in order. As ever, this should ideally be supported by clinical trial data, which is notably lacking in the population of interest. We would suggest that a large trial of different treatment regimens with standardized microbiological outcomes and short follow-up could be achieved relatively quickly and cheaply in an area highly co-endemic for acute malnutrition and helminthiasis.

In terms of the more fundamental question of whether empiric treatment is warranted in the absence of clear clinical or laboratory indications, a pragmatic trial scheduled to start in January 2013 in Malawi will provide the first clear evidence of its value in the management of SAM, by comparing single dose albendazole to placebo alongside standard current management (NCT01395381).

## SKIN INFECTIONS

Children with SAM frequently have areas of skin erosion or breakdown, thought to be related to zinc or other micronutrient deficiencies that are addressed by therapeutic feeding. This is particularly the case in kwashiorkor, where a characteristic dermatosis is seen over pressure points, areas of oedema, and around the perineum. As well as being uncomfortable and distressing in their own right, such dermatoses represent potential portals of entry for pathogenic microorganisms including bacteria and candida species. Severe skin disease is a complication that may require inpatient care.

### How should children with SAM and evidence of skin or soft tissue infection be treated?

Skin infections take a variety of forms:

- They may be superficial and localised to the skin surface itself (impetigo).
- They may be localised but affecting the surrounding skin and soft tissues with fever, swelling and spreading erythema, potentially leading to infection of deeper structures, which may be very severe.
- They may be invasive, with features of systemic infection and haematogenous spread of the causal organism to distal sites like bone or meninges.

Localised infections can lead directly to invasive disease and all visible evidence of skin or soft tissue infection (SSTI) such as redness, swelling or fever in the context of SAM should be regarded as complicated SAM and treated in hospital with IV antibiotics. In this case, a penicillinase-stable penicillin (e.g. cloxacillin) should be provided alongside ampicillin and gentamicin in order to provide strong coverage against *Staphylococcus aureus*. It is important that this is provided *alongside* rather than *instead of* ampicillin, because cloxacillin does not have the same activity against Gram negative microorganisms as ampicillin. These should also be considered as potential pathogens, especially where skin breakdown occurs around the nappy area.

In that invasive infection arising from skin breakdown may occur even without clinical specific features of SSTI (i.e. localizing features), we provide cloxacillin as an additional first line antibiotic if breakdown is present and the child seems particularly unwell. Otherwise it is reasonable to monitor response to treatment with ampicillin and gentamicin, and switch to ceftriaxone (which has good activity against *Staph. aureus*) if required. The one proviso to this is that where there is very strong clinical suspicion of *Staph. aureus* infection (or a positive blood culture), cloxacillin is actually a more effective antimicrobial choice than ceftriaxone.

### What care should be given for areas of skin breakdown?

Careful care of wounds or areas of skin breakdown is essential both to encourage their healing, and to prevent the development of infectious complications. The regimen outlined by WHO involves the following:

- Potassium permanganate solution (0.01%): daily bathing.
- Gentian violet or nystatin cream: applied to ‘skin sores’.
- Keeping the area clean and dry by use of barrier creams (zinc and castor oil ointment, or petroleum jelly, or tulle gras (bandages impregnated with paraffin oil)) and omitting nappies.^6^

There is no clinical trial evidence on which to judge the adequacy of such a regimen, and it is highly likely that the level of care taken by nursing or medical staff and carers in looking after the skin is more important that the fine details of which agents are used. That said, while potassium permanganate is used both for astringent (drying) and antiseptic activities and provides antisepsis against a broad range of microorganisms, gentian violet and nystatin are predominantly active against Gram +ve bacteria and yeasts (respectively).^204^ Theoretically, an antiseptic agent that has activity against Gram –ve bacteria as well, would be preferable, especially where there is perineal involvement. Such agents include chlorhexidine, povidone-iodine, and silver sulfadiazine. Where local candidiasis is suspected, a topical antifungal cream such as nystatin should certainly be included. When dispensing creams, it is critical that rigorous hand-hygiene is observed. Where possible, individual pots of cream should be used for each patient (ideally closed with a pump dispenser): the use of communal pots is an entirely avoidable risk for the spread of resistant microorganisms amongst these vulnerable patients.

## ADDITIONAL RESOURCES ON PHARMACOLOGY IN SAM

The pharmacology of antimalarials in SAM is reviewed in annex 3 of the latest WHO guidelines for the management of malaria: http://helid.digicollection.org/pdf/s13418e/s13418e.pdf.

Physiological factors affecting drug disposition in relation to malnutrition have been reviewed here: http://www.ncbi.nlm.nih.gov/pmc/articles/PMC2794862/.

A systematic review of pharmacokinetics in malnutrition is published here: www.ncbi.nlm.nih.gov/pubmed/20552179.

**Table:** Box 1 Example of misclassification of nutritional status due to dehydration. A fourteen-month-old boy presents with a history of AWD, low urine output in the past 24 hours, and of drinking poorly – he is now lethargic and unable to drink at all. He is assessed by a clinician who finds him to be severely dehydrated with features of shock, and severely malnourished (on the basis of weight-for-length z-score = −3.16). Following WHO guidelines, the child receives two boluses of 15 ml/kg IV fluids over the first two hours (responding well to this), and then ORS via nasogastric tube overnight (at 10 ml/kg/hour) for 8 hours followed by maintenance F-75. In the morning the child looks much better, and is no longer dehydrated. He has received a total of 120 ml/kg fluids for rehydration followed by maintenance. His anthropometry has changed as below: The weight at presentation was 11% lower than his weight after rehydration and WLZ suggests he was not acutely malnourished. His MUAC still indicated MAM, so he was referred to a supplementary feeding programme on discharge.

	Before resuscitation	After resuscitation
Weight (kg)	7.6	8.5
Length (cm)	79.3	79.3
Mid-upper arm circumference (cm)	11.6	12.2
Weight-for-age z-score	−1.77	−0.73
Length-for-age z-score	1.07	1.07
Weight-for-length z-score	−3.16	−1.69
